# Shell microelectrode arrays (MEAs) for brain organoids

**DOI:** 10.1126/sciadv.abq5031

**Published:** 2022-08-17

**Authors:** Qi Huang, Bohao Tang, July Carolina Romero, Yuqian Yang, Saifeldeen Khalil Elsayed, Gayatri Pahapale, Tien-Jung Lee, Itzy E. Morales Pantoja, Fang Han, Cynthia Berlinicke, Terry Xiang, Mallory Solazzo, Thomas Hartung, Zhao Qin, Brian S. Caffo, Lena Smirnova, David H. Gracias

**Affiliations:** ^1^ Department of Chemical and Biomolecular Engineering, Johns Hopkins University, Baltimore, MD 21218, USA.; ^2^ Department of Biostatistics, Bloomberg School of Public Health, Johns Hopkins University, Baltimore, MD 21287, USA.; ^3^ Center for Alternatives to Animal Testing, Department of Environmental Health and Engineering, Bloomberg School of Public Health and Whiting School of Engineering, Johns Hopkins University, Baltimore, MD 21205, USA.; ^4^ Department of Civil and Environmental Engineering, Syracuse University, Syracuse, NY 13244, USA.; ^5^ Department of Statistics, University of Washington, Seattle, WA 98195, USA.; ^6^ Department of Ophthalmology, Johns Hopkins University School of Medicine, Wilmer Eye Institute, Baltimore, MD 21287, USA.; ^7^ CAAT-Europe, University of Konstanz, 78464 Konstanz, Germany.; ^8^ Environmental Metrology & Policy Program, Georgetown University, Washington, DC, 20057, USA.; ^9^ Department of Molecular Microbiology and Immunology, Bloomberg School of Public Health, Johns Hopkins University, Baltimore, MD, 21205, USA.; ^10^ Department of Materials Science and Engineering, Johns Hopkins University, Baltimore, MD 21218, USA.; ^11^ Department of Chemistry, Johns Hopkins University, Baltimore, MD 21218, USA.; ^12^ Sidney Kimmel Comprehensive Cancer Center, Johns Hopkins School of Medicine, Baltimore, MD 21205, USA.; ^13^ Laboratory for Computational Sensing and Robotics (LCSR), Johns Hopkins University, Baltimore, MD 21218, USA.; ^14^ Department of Oncology, Johns Hopkins University School of Medicine, Baltimore, MD 21205, USA.

## Abstract

Brain organoids are important models for mimicking some three-dimensional (3D) cytoarchitectural and functional aspects of the brain. Multielectrode arrays (MEAs) that enable recording and stimulation of activity from electrogenic cells offer notable potential for interrogating brain organoids. However, conventional MEAs, initially designed for monolayer cultures, offer limited recording contact area restricted to the bottom of the 3D organoids. Inspired by the shape of electroencephalography caps, we developed miniaturized wafer-integrated MEA caps for organoids. The optically transparent shells are composed of self-folding polymer leaflets with conductive polymer–coated metal electrodes. Tunable folding of the minicaps’ polymer leaflets guided by mechanics simulations enables versatile recording from organoids of different sizes, and we validate the feasibility of electrophysiology recording from 400- to 600-μm-sized organoids for up to 4 weeks and in response to glutamate stimulation. Our studies suggest that 3D shell MEAs offer great potential for high signal-to-noise ratio and 3D spatiotemporal brain organoid recording.

## Teaser

Miniaturized electrode caps are fabricated and used for 3D electrical recording from brain organoids.

## INTRODUCTION

As direct research on the human brain has been practically and ethically limited and animal models have limitations of interspecies differences, in vitro models using human cells have emerged as an attractive alternative approach to understanding neuronal circuitry, neurotoxicity, neurological disorders, and brain development (*1*–*3*). Notably, there has been remarkable progress in the field of human in vitro models in recent years (*4*, *5*). In particular, pluripotent stem cell–derived brain organoids, with their three-dimensional (3D) multicellular architecture and development profile, have been shown to replicate key features of the human brain (*6*–*11*). Concurrent with the development of brain organoids is the need to develop electronic and optical infrastructure for in situ stimulation and recording of electrical activity to assess their functionality and physiological relevance.

Multielectrode arrays (MEAs) provide noninvasive and high-speed recording and network mapping of extracellular electric field potential (*12*–*14*). However, traditional in vitro MEA plates use predominantly planar electrode interfaces, initially designed for monolayer cultures, thus limiting the contact surface area with 3D organoids (*3*, *15*). Several modifications, such as spine-shaped electrodes, nanowires, and 3D nanostructures, have been patterned on these MEA plates to increase the signal. For example, spine-shaped gold electrodes have been used to reduce the extracellular cleft for a better signal-to-noise ratio (SNR) (*16*), and vertical nanowires provide recording access to the interior of the measured cell via penetration (*17*). Nevertheless, even in such devices, the recording contact area would still be limited to the bottom of the organoid where it is attached to the plate (*16*–*19*). Recently, several curved and folded shapes have been introduced for MEA recording, including buckled, cylindrical, and shells (*20*–*26*). For example, we have previously used SiO/SiO_2_ bilayers to create a self-folding shell for single/few cardiac cell encapsulation and measurement, but the small dimensions and rigid material composition of such single-cell devices may not be scalable and suitable for larger brain organoids (*20*). Kalmykov *et al.* (*21*, *23*) have developed a cylindrical sensor array for cardiac and cortical organoids. This methodology requires manual unrolling of the cylinder with a micromanipulator to enclose the organoids, which can limit throughput and also has a limited cylindrical shape. Park *et al.* (*24*) have demonstrated a buckled mesoscale spheroid neuro interface for brain organoid recording. This approach requires a prestrained elastomeric substrate that can limit integration with other silicon modules or microfluidic devices.

Here, we report a silicon wafer–integrated self-folding polymer shell MEA platform for brain organoids. We developed a wafer-scale microfabrication process to create shell MEAs by using a self-folding polymer bilayer (*27*). The fabrication process is straightforward, making it potentially compatible with MEA plates, tunable, scalable, and cost-effective. The essential element is a self-folding negative photoresist polymer (SU8) bilayer with tunable folding based on the relative thickness and exposure energy. Gold wires and contact pads are integrated within the self-folding bilayer for good insulation with exposed conductive polymer poly-(3,4-ethylenedioxythiophene):poly (styrene sulfonate) (PEDOT:PSS)–coated electrodes. The extent of self-folding was guided by a finite element method (FEM) model, with the constitutive relations and volume shrinkage after acetone treatments developed for SU8 cross-linked by different exposure energy. We show that we can create customizable shells for organoids of different sizes, creating a loose or firm contact between the electrodes and the brain organoids. The materials and self-folding processes are biocompatible, and the transparent leaflets of the shell electrodes allow for in situ bright-field and fluorescence imaging. We demonstrate 3D spatiotemporal recording of brain organoids encapsulated in the 3D shell electrodes with and without glutamate stimulation. We contrast 2D and 3D recordings by comparing cumulative firing and electrical responses to glutamate. The firing was defined via a simple threshold, and spike distributions were compared nonparametrically using a Wilcoxon rank sum (permutation) test. The responses to the glutamate intervention were summarized using the nonparametric Mann-Kendall trend test, and the inference was again performed by permuting MEA type (2D and 3D) labels. Both a distributional shift in firing with a higher firing rate and a stronger estimated glutamate-mediated trend were detected using 3D MEAs. Together, our platform and studies demonstrate a distinct 3D methodology for mapping electrical activity in brain organoids, offering a larger recording contact area as compared to conventional MEAs.

## RESULTS

### Concept and fabrication of the shell MEAs

**Fig. 1.**
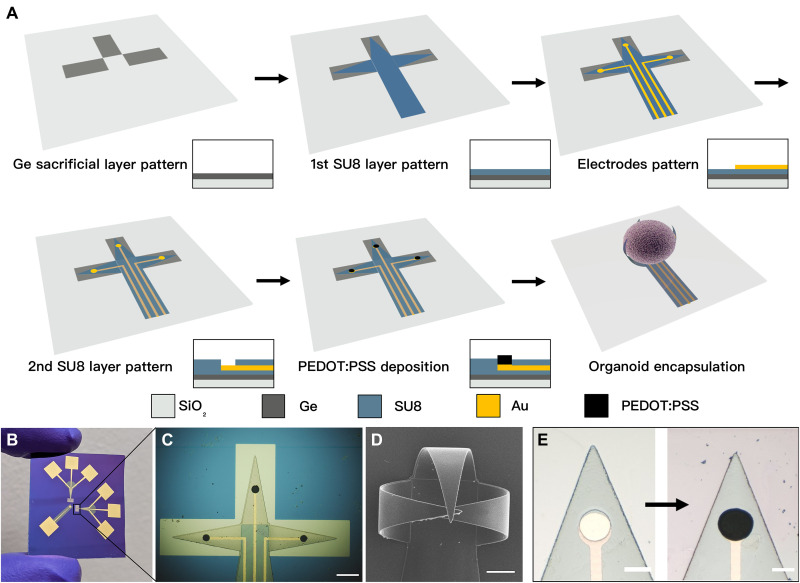
Fabrication process flow and experimental images of the 3D shell MEAs. (**A**) Fabrication process flow of 3D shell MEAs. (**B**) An optical image of the fabricated shell MEAs. (**C**) A zoomed-in optical image of the shell electrodes in the flat state. Scale bar, 200 μm. (**D**) Scanning electron microscopy (SEM) image of the shell MEAs after actuation. Scale bar, 100 μm. (**E**) The recording electrodes on the leaflet before (left) and after (right) conductive polymer PEDOT:PSS electroplating. Scale bar, 50 μm.

Our development of 3D shell electrodes was inspired by macroscale electroencephalography (EEG) caps that are used to study the electrical activity of the human brain (*28*). These caps are typically composed of flexible materials with multiple metal electrodes covering the entire scalp, allowing sampling of electrical signals from all over its 3D shape. Likewise, we designed our shell MEAs to consist of leaflets that can wrap around the surface of the brain organoids. As a proof of concept, we patterned three leaflets with three electrodes distributed on the 3D surface. Our fabrication approach is compatible with alternate designs containing arrays of multiple electrodes and leaflets folding at different angles (*27*). Briefly, our fabrication process involved the following steps: (i) deposition of sacrificial layer, (ii) patterning of the first SU8 layer, (iii) patterning of gold wiring, (iv) patterning of the second SU8 layer, (v) patterning of the PEDOT:PSS electrodes, (vi) dissolution of the sacrificial layer and preconditioning, (vii) sterilization and placement in cell medium, and (viii) organoid placement and self-folding as depicted in Fig. 1A. Our current process used five photomasks, and we typically fabricated eight shells with three electrodes each on a 3-inch silicon (opaque, SiO_2_ coated) or quartz (transparent) wafer (Fig. 1B). We chose SU8 for our shell MEAs since it is a popular negative photoresist and has been already widely used in microfluidics and microelectromechanical system processes (*29*). We have previously reported the fabrication process and demonstrated the self-folding mechanism of 3D SU8 architectures with solvent exchange (Fig. 1, C and D) (*27*). Because of its widespread use in microsystem fabrication, there is an abundance of technical knowledge on its processing, biocompatibility, and ease of integration with other modules that may be integrated in the future for chemical or optical interrogation of organoids (*30*). We chose gold for the wiring because of its high electrical conductivity and good biocompatibility. We added a conductive polymer PEDOT:PSS coating on the electrode to reduce impedance and minimize the modulus mismatch for the MEAs/organoid contact (Fig. 1E and fig. S1) (*19*, *31*). A reference electrode was placed near the shell electrodes for data acquisition (fig. S2). Our fabrication and folding process is compatible with AutoCAD mask design and multilayer photolithography and, as a result, scalable.

### Brain organoid characterization

**Fig. 2.**
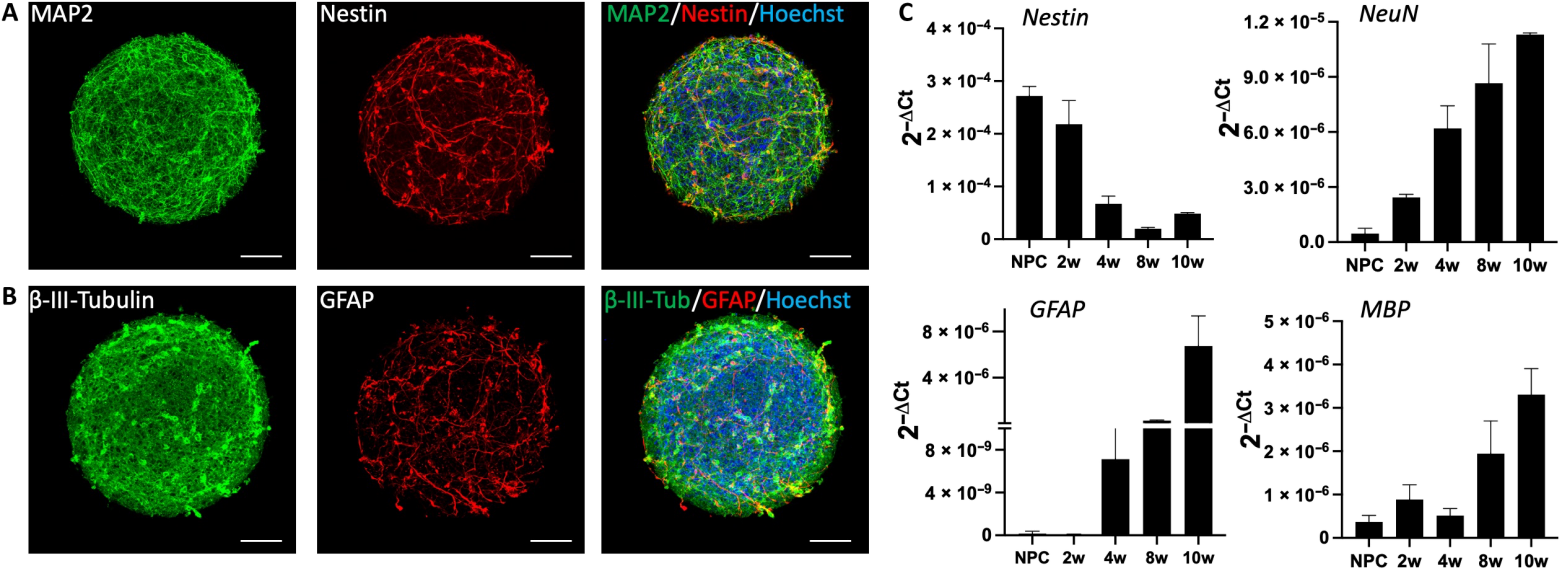
Brain organoid model. (**A**) Eight-week brain organoid stained with neuronal marker MAP2 (green) and neuroprogenitor marker, Nestin (red). (**B**) Eight-week brain organoid stained with neuronal marker β-III-tubulin (green) and astrocyte marker, GFAP (red). Nuclei are stained with Hoechst (blue). Scale bar, 100 μm. (**C**) RT-PCR showing expression of neuroprogenitor (*Nestin*), neuron (*NeuN*), astrocyte (*GFAP*), and oligodendrocyte (*MBP*) genes over the course of 10 weeks of differentiation. Data are shown as means ± SEM, *n* = 3. MAP2, microtubule-associated protein 2; GFAP, glial fibrillary acidic protein; MBP, myelin basic protein; NeuN, neuronal nuclei; NPC, neuroprogenitors; 2w, 4w, 8w, and 10w indicate 2, 4, 8, and 10 weeks of differentiation starting from NPC stage, respectively.

We generated the brain organoids used in this study from human induced pluripotent stem cells (iPSCs, NIBSC8 line). We differentiated neuroprogenitor cells (NPCs) (time zero) and then brought an NPC cell suspension to form 3D brain organoids under constant gyratory shaking for up to 8 to 10 weeks, as previously described (*8*). Mature brain organoids are homogeneous, ranging from 400 to 600 μm, and consist of neurons, astrocytes, and oligodendrocytes (Fig. 2).

In Fig. 2 (A and B), we show an 8-week brain organoid stained with neuronal markers microtubule-associated protein 2 (MAP2) and β-III-tubulin (green), the neuroprogenitor marker Nestin (red), and the astrocyte marker glial fibrillary acidic protein (GFAP) (red). Figure 2C shows gene expression of neural markers of differentiation over time, covering stages of NPCs to 10 weeks of differentiation. The maturation over time resulted in an increase in mature neuronal [neuronal nuclei (NeuN)], astrocyte (*GFAP*), and oligodendrocyte [myelin basic protein (*MBP*)] markers and a decrease in neuroprogenitor markers (*Nestin*). The presence of mature neurons, astrocytes, and mature oligodendrocytes is key to the brain organoid functionality, which is measured by the system’s electrical activity. Maturation of neurons provides increased synaptic formation, while astrocytes support this process. Oligodendrocytes provide myelin sheaths around the axons, which reduce the ion leakage and capacitance of the cell membrane for efficient electrical signal transmission (*32*, *33*). We previously demonstrated that following our protocol, we have up to 40% of myelinated axons (*8*), which are known to be challenging to model in vitro with human brain cells.

### Tunability of fold angle, shape, and electrode pattern

We combined numerical simulation and experimental trials to develop a rational and reliable design for self-folding to enable reproducible assembly and ensure good contact between the organoids and shell MEA electrodes. An optimal shell MEA device should fit the outer contour of the target brain organoids like a cap (fig. S3). The critical parameters that control fold angle include SU8 bilayer thickness and ultraviolet (UV) exposure.

**Fig. 3.**
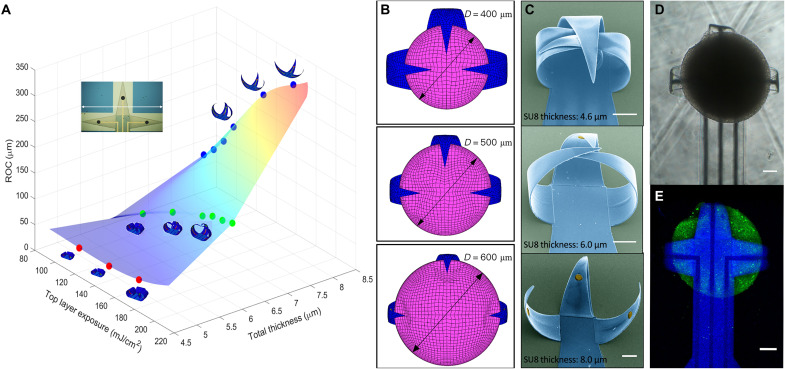
FEM simulation of the programmable folding of 3D MEAs and optical images of brain organoid encapsulation. (**A**) Plot depicting the simulated radius of curvature (ROC) as a function of the SU8 bilayer thickness and the top layer exposure energy. The top left image shows the size of the shell in flat state. The tip-to-tip distance from left to right is 1450 μm. (**B**) FEM snapshots showing organoids of different sizes (400 to 600 μm) fitting in tailored shell electrodes. (**C**) Corresponding SEM images of 3D shell electrodes with different levels of folding. The images are false-colored, with blue indicating the SU8 shell and yellow indicating the electrodes. Scale bar, 100 μm. (**D**) Bright-field image of the organoid in a 3D shell MEA, and (**E**) confocal image showing the top view (projected confocal stack) of a brain organoid (green, Fluo-4 calcium dye) with a diameter around 500 μm encapsulated in the 3D shell (blue) electrodes. Scale bar, 100 μm.

We developed a FEM model and used it to simulate the self-folding behavior of the polymer shell together with the electrodes (details in notes S1 and S2). This model allows us to independently tune the design parameters, including the overall size and geometry, the bilayer/electrode thickness, and UV exposure of the top and bottom layers, and predict the fully equilibrated structures after folding in simulations. For typical shell MEAs presented in Fig. 1C, we ran simulations to compare experimental results and study the effects of SU8 bilayer thickness and the exposure energy difference between the two layers on the final folded shell shape (Fig. 3A). Overall, the simulation results clearly demonstrate that the folding increases (i.e., smaller radius of curvature) as the thickness decreases. As we expanded the thickness of the SU8 bilayer, the folding decreased because of higher bending stiffness more than the countereffect caused by the mismatch strain in the two layers. Furthermore, the exposure energy difference between the SU8 bilayers also played an important role in the final desired 3D shape of the shell electrode. As the exposure energy of the top layer increases, the overall folding decreases. The exposure energy difference between the SU8 bilayers generates an overall polymerization gradient across the bilayer. The polymerization gradient causes a mismatch strain along the bilayer thickness and provides a bending moment for the SU8 bilayer to fold spontaneously. We can rationally predict the folding by FEM and, in principle, produce 3D shell architectures from the 100- to 10-cm scale with our lithographic integration process (*27*). This large range of size tunability is advantageous for recording activities from organoids of different sizes.

We compared the FEM results with the experimental measurements by using three conditions of the SU8 bilayer with different thicknesses and top layer UV exposure (from top to bottom: 4.6 μm, 180 mJ/cm^2^ UV exposure; 6.0 μm, 180 mJ/cm^2^ UV exposure; and 8.0 μm, 120 mJ/cm^2^ UV exposure), and the bottom layers were fully cross-linked (240 mJ/cm^2^ UV exposure). These different conditions yielded bilayers folding to different extents that agreed with the simulation results (Fig. 3C). Next, we investigated how the shell electrodes captured brain organoids of varied sizes. In general, the brain organoids used here had diameters between 400 and 600 μm. Thus, we simulated how the different shell electrodes encapsulated the brain organoids with 400-, 500-, and 600-μm diameters, respectively (Fig. 3B). These simulation results matched with our experimental observations, where the brain organoids were well encapsulated in the 3D folded shell electrodes (Fig. 3D). A confocal microscope calcium image of a brain organoid enveloped inside the 3D folded shell electrodes is shown in Fig. 3E (*34*). The transparent SU8 leaflets of the 3D shell electrode device offer the potential for future optical stimulation. The time scale of the folding process of the 3D shell electrodes (typically complete within an hour) includes adequate time for sterilization and encapsulation of the brain organoids (fig. S4). The folding of the shell electrodes is reversible with the solvent exchange, which showed the potential for reusable electrodes (fig. S5). A cytotoxicity test was performed on the encapsulated brain organoid and compared with the results of the free-floating organoid (fig. S6) to verify the biocompatibility of the process.

### Feasibility of spontaneous activity and glutamate-induced electrical activity recording

**Fig. 4.**
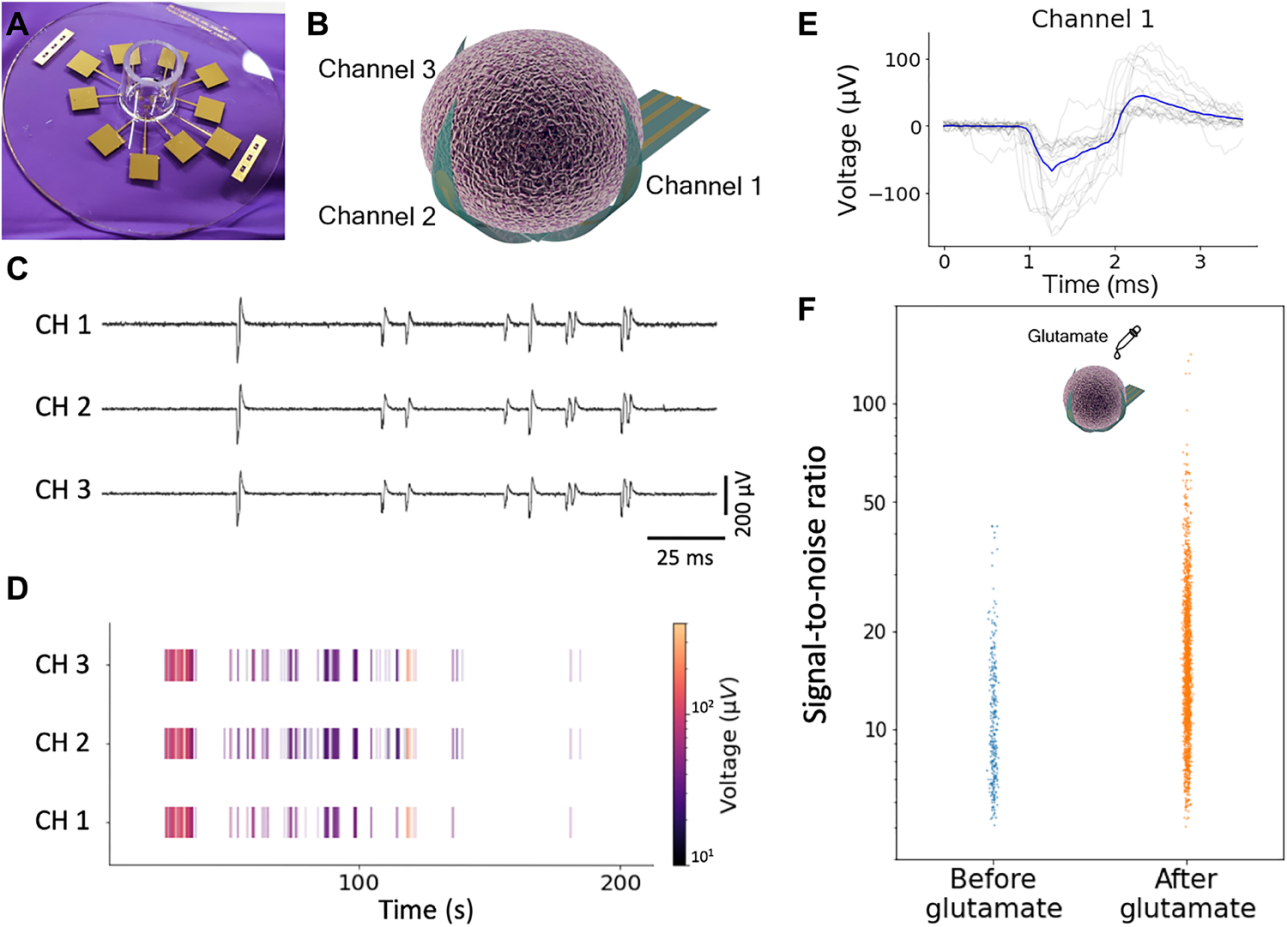
3D shell MEA recordings from encapsulated brain organoids. (**A**) Image of a quartz wafer–integrated 3D shell MEA. (**B**) Electrode distribution of the 3D shell MEA around the brain organoid. (**C**) Typical field potentials recorded from a brain organoid encapsulated within a 3D shell MEA. CH, channel. (**D**) Representative raster plot of the spontaneous firing of the brain organoid. (**E**) Representative overlaid spike waveform from channel 1. (**F**) Comparison of spike distribution from the recorded brain organoid before and after glutamate treatment.

We demonstrate the feasibility of recording 3D spatial electrophysiological activities of the brain organoid across the electrodes on each leaflet. A 9-week brain organoid was placed at the center of the shell electrodes during self-folding, which allowed the organoid to be enveloped within the leaflets with the PEDOT:PSS electrodes in intimate contact with the organoid (Fig. 4B). A glass cylinder was placed around the device to hold the cell medium. Electrode outputs were fabricated for signal acquisition (Fig. 4A). The three PEDOT:PSS electrodes on the leaflets successfully recorded the field potential of the enveloped brain organoid (Fig. 4C and fig. S7). The raster plot (Fig. 4D) shows the recording of the spontaneous activity of the enveloped brain organoid (*35*, *36*). The overlaid spike waveform indicates an average spike duration of ~2 ms, with varied amplitude during the recording (Fig. 4E). We note that we can add more electrodes to the leaflets to improve the recording (figs. S8 and S15).

We further confirm that the signal recorded is from the brain organoids by examining the response to 20 μM glutamate neurotransmitter added to the culture medium that served as a positive control for the spontaneous recording (*23*, *37*). The spike SNR before and after the glutamate application showed statistically significant amplification (median increased by 57.6%) on the amplitude of firing spikes (Fig. 4F). A detailed clustering also demonstrated that longer interspike interval was observed in the glutamate-induced signals (fig. S9).

### Design of different electrode configurations

To gain insight into the relative merits of 3D versus 2D recording and proximal versus distal electrodes, we designed an array that features four electrodes on the bottom surface in addition to the 3D shell MEA electrodes. This configuration also demonstrates the ease with which our fabrication methodology can be tuned for different electrode combinations.

While the conventional MEA recording methodology is well established, the recording from 3D multielectrode devices is still at a very early stage. In previous studies, researchers have compared 3D recordings with conventional 2D recordings, but these are typically different organoids (*24*). We also carried out brain organoid recording using a 2D MEAs system (Axion BioSystems; fig. S10) and from our shell MEAs in an unfolded format (fig. S11). Both showed similar spike duration of approximately 2 ms. These studies suggest the feasibility of our recording platform to perform recordings in 2D and allow us to compare these 2D recordings with the 3D recordings.

Of note, the 3D MEAs offer more recording electrode pads with greater contact area and spread around the organoid. To directly observe the influence of these features, we designed 3D shell electrodes with three electrodes, one on each leaflet and an additional fourth electrode on the bottom center of the shell (fig. S12A). Once the shell electrodes were folded and the organoid was encapsulated (fig. S12B), we were able to record not only from the 3D folded shell electrodes but also from the bottom electrode. We observed that the cumulative spike counts from the three folded electrodes were significantly larger than that from the bottom electrode alone (fig. S12C). While it is mathematically necessary that the union of spike events is larger than the events from a single electrode, fig. S12 demonstrates the scope of the difference. We also observed signal heterogeneity between different electrodes, indicating that such MEAs could probe spatiotemporal electrical activity (fig. S12D). Organoid 1 is the most extreme since most spiking occurred away from the bottom electrode. The stacked bar chart (fig. S12C) shows that electrodes 1, 2, and 3 bring independent information that is lost if only the bottom electrode is considered.

**Fig. 5.**
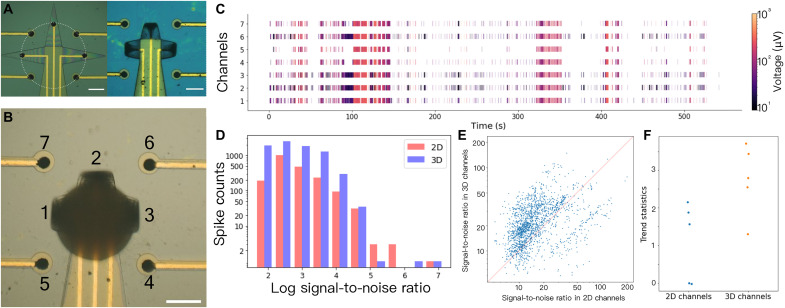
Investigation of different electrode configurations. (**A**) Optical images of the 3D shell electrodes and 2D electrodes in flat (left) and self-folded (right) state. Scale bar, 200 μm. (**B**) Optical image of the 3D shell electrodes (nos. 1, 2, and 3) encapsulating the brain organoid, along with the 2D electrodes (nos. 4, 5, 6, and 7). Scale bar, 200 μm. (**C**) Raster plot of the 3D shell and 2D recordings. (**D**) Histograms of spike counts having different signal-to-noise ratios (SNRs), comparing 3D and 2D channels. The graph displays that the 3D channels have a greater capacity to capture signals with different SNRs. (**E**) The SNR of the paired spikes recorded by 3D and 2D electrodes. (**F**) Trend statistics of the 2D and 3D channels with glutamate stimulation.

With our shell electrode methodology, we can fold distal electrodes into proximal electrodes with 3D spatial distribution. Because of the sample variation across brain organoids, a systematic study on the difference between the recording from 2D (prefolding) electrodes and 3D (folded) shell electrodes from the organoids is challenging and has been lacking in the published literature. Hence, we designed an electrode system and performed experiments in which we have both the 2D electrodes (nos. 4, 5, 6, and 7 in Fig. 5A) and 3D shell electrodes (nos. 1, 2, and 3 in Fig. 5A) able to record signals simultaneously from the same brain organoid. This also allowed us to investigate the effect of distance between the electrode and brain organoid. Before the folding process, all the electrodes were equidistant to the center of the flat shell (Fig. 5A). Once the MEA shell folded, the leaflets encapsulated the brain organoid so that specific electrodes were proximal to the brain organoid (Fig. 5B), while others were not. Achieving such localization can be difficult for conventional MEAs since the location of the brain organoids seeded on 2D MEAs cannot be well controlled as they are not encapsulated, and the only proximal electrode location is the exact bottom of the spherical organoid.

We note that in 2D MEA recordings, a cell adhesive protein or Matrigel coating is needed to enhance attachment and promote neurite grown from the organoid to electrodes, while no coatings are needed in the shell MEAs. The 3D shell MEA secures the organoid within its grasp and keeps it relatively stable as compared to 2D MEAs during motion with the electrodes proximal to the organoid. In addition, the absence of a surface coating in 3D MEAs prevents individual cell migration from the organoids and neurite outgrowth, which is unavoidable in 2D MEAs, where a coating is essential to keep organoids attached to the electrodes. Thus, in 2D MEAs, even distant electrodes will be in touch with unknown number of cells and neurites, leading to higher variability in recordings. In our experiments (Fig. 5A), the 2D distance electrodes were not in contact with the cells or neurites. We recorded the field potentials from the four 2D electrodes and three 3D electrodes, as shown in the raster plot (Fig. 5C and fig. S13). With five rounds of spontaneous recording from three different brain organoids, we detected 7785 spikes from the 3D shell electrodes and 2025 spikes from the 2D planar electrodes. The results indicate that as the 3D shell electrodes folded up to reach the surface of the 3D organoid, they could detect more spikes than the distanced electrodes from the brain organoid (Fig. 5D). Furthermore, to conduct a direct and fair comparison of the recording quality, we analyzed the 1860 spikes detected both by the 3D shell electrodes and by the 2D planar electrodes. The SNR of the same spikes in the 3D shell electrodes channels was significantly higher (yielding a 42% increase in the median, *P* < 0.005) than in the 2D channels (Fig. 5E). We also compared the sensitivity between 3D and 2D recording in response to glutamate stimulations (fig. S14). We stimulated the brain organoid for five rounds with 20 μM glutamate, and the 3D shell electrodes detected a significantly stronger increase in electrophysiological activities (quantified by Mann-Kendall’s *z* of spike amplitudes, *P* = 0.02; Fig. 5F) (*38*). In summary, for brain organoids, the recording from 3D shell electrodes detected more spikes and were more sensitive to stimulation-induced activities, thus augmenting the electrophysiological recordings compared to the original 2D electrodes with a potential for spatial analysis in the future.

## DISCUSSION

In summary, we have reported a wafer-integrated self-foldable 3D shell MEA neural interface for brain organoids. We have created both a rational design and customizable fit for organoids of different sizes, which is important for recording from organoids during different stages of maturation and development. The inclusion of polymer (SU8 and conductive PEDOT:PSS coatings) as compared to significantly more rigid silicon or metallic materials minimizes modulus mismatch between the recording device and the organoid (*39*). Our differentially cross-linked bilayer self-folding approach could also be used with even softer polymers and hydrogels in future iterations (*40*). Our self-folding process is highly parallel, compatible with conventional photolithography, and does not require any probes or transfer steps. Hence, it can be implemented at wafer scale for facile microintegration with alternate microelectronic, microfluidic, and micromechanical components. We can also readily vary the spacing and layout of recording electrodes and wires using conventional lithographic processes, which is necessary to increase inputs/outputs (I/O). MEAs can be used for stimulation in addition to recording. Furthermore, our integration approach is amenable to the integration of complementary metal-oxide semiconductor (CMOS) or related components. We have demonstrated robust 3D recording from our 3D shell electrodes and evaluated their performance relative to 2D electrodes and in the presence of stimulants.

In the future, we will seek to address some of the limitations of the present study. For example, in the current study, we needed to manually insert the organoids into the shell electrodes. In addition, with integration of microwell or microfluidic interfaces, we anticipate that we can grow the organoids inside the shell electrodes so that we can form high-throughput arrays of electrode-integrated organoids (*26*, *41*). On the basis of the current design, we can also increase the number of I/O or use CMOS sensors for higher recording resolution by using other interconnect layouts and higher-resolution lithography, and create porous leaflets for enhanced oxygen and nutrient transportation, and stable long-term recording and stimulation. In addition, it is conceivable that foldable electrodes with surface patterns or protrusions can be used so that they penetrate and facilitate recording from within the organoid, and these strategies are compatible with our microfabrication and self-folding approach (*22*).

This study opens avenues for more in-depth analysis of the brain organoid connectome of neural cells and brain organoid to machine interfaces. Artificial intelligence–based analysis methods of human EEGs (*42*, *43*) lend themselves for the analysis of such recordings. Ultimately, such analysis might allow the realization of primitive cognitive functions in brain organoids.

## MATERIALS AND METHODS

### Fabrication and actuation of 3D shell electrodes

First, we patterned a 50-nm-thick germanium (Ge) sacrificial layer on either a SiO_2_ or quartz (transparent) wafer. Then, we spin-coated a layer of SU8 2002 or 2005 (Kayaku, Westborough, MA) on top of the substrate, and we patterned and fully cross-linked the first layer of SU8 via photolithography through a photomask and developed in SU8 developer for 1 min to define the shape of the first layer. On top of the first SU8 layer, we spin-coated a ~2.7-μm Shipley SC 1827 photoresist (Kayaku, Westborough, MA). We defined patterns of electrodes using photomasks followed by 1-min development in 351 Developer (1:5) (Kayaku, Westborough, MA). We deposited the electrodes (Cr, 10-nm adhesion promoter; Au, 50 nm) using thermal evaporation and liftoff. We patterned the bilayer of SU8 on top of the electrode layer, partially cross-linked, thus forming an SU8 solvent-responsive bilayer and electrically insulating the electrodes. We used SU8 2005 at 3000 rpm for both SU8 layers to get a bilayer with a thickness of 8.0 μm; we used an SU8 mixture (25% SU8 2005 and 75% SU8 2002) for both SU8 layers to get a bilayer with a thickness of 6.0 μm; we used an SU8 mixture (50% SU8 2005 and 50% SU8 2002) for both SU8 layers to get a bilayer with a thickness of 4.6 μm.

### Electroplating of the conductive PEDOT:PSS electrode coating

We prepared the PEDOT:PSS electrolyte by mixing 10 mM EDOT (Sigma-Aldrich, St. Louis, MO) and PSS (Sigma-Aldrich, St. Louis, MO) 0.4 wt % in deionized (DI) water. After cleaning the wafer with acetone and oxygen plasma, we connected the electrode pads with copper wires using alligator clips. We then attached the working electrode (Pt) to the anode and the sample to the cathode for electroplating. We set the current density at 12.5 mA/cm^2^ and deposited the PEDOT:PSS for 4 min to create a 10-μm-thick coating. After electroplating, we rinsed the sample with DI water, and a dark blue PEDOT:PSS coating can be seen on top of the Au electrode layer.

### Characterization of PEDOT:PSS coatings

The impedance of PEDOT:PSS coatings was measured using an Intan RHD recording system (Intan Technologies, Los Angeles, CA) in 1× phosphate-buffered saline (PBS) at 1000 Hz. The height profile was acquired using a Keyence laser scanning microscope VK-X100.

### Actuation of 3D shell electrodes

We dissolved the Ge sacrificial layer in 5% hydrogen peroxide. Once the Ge sacrificial layer disappeared, we immersed the flat shell electrodes in acetone for 5 min to precondition (remove any uncross-linked SU8) the folding of the shell electrodes. We subsequently placed the 3D shell electrodes back in the water and rinsed them three times. We actuated the self-folding of the shell MEAs by placing back in aqueous solutions.

### Brain organoids

We differentiated brain organoids from the iPSC NIBSC8 cell line [U.K. National Institute for Biological Standards and Control (NIBSC)], following our in-house two-step protocol (*8*). The NIBSC-8 iPSC cell line is mycoplasma free, with a normal female karyotype. Briefly, we differentiated iPSCs in a monolayer to NPCs using serum-free, chemically defined neural induction medium (Gibco, Thermo Fisher Scientific). We expanded the NPCs, and a single-cell suspension was distributed into uncoated six-well plates and cultured under constant gyratory shaking (80 rpm, 19-mm orbit) to form 3D aggregates. After 48 hours, we induced differentiation with serum-free, chemically defined differentiation medium [Neurobasal electro medium (Gibco, Thermo Fisher Scientific) supplemented with 1× B27-electro (Gibco, Thermo Fisher Scientific), 2× glutamax, glial cell line–derived neurotrophic factor (10 ng/ml; Gemini), brain-derived neurotrophic factor (10 ng/ml; Gemini), and 5% penicillin-streptomycin]. We differentiated brain organoids for 8 to 10 weeks before recording. By this time, the brain organoids consist of different types of neurons, astrocytes, and oligodendrocytes (*8*).

### Immunohistochemistry

We fixed the brain organoids at 8 weeks of differentiation with 2% paraformaldehyde for 45 min, blocked with 10% goat serum, 1% bovine serum albumin (BSA), and 0.15% saponin in 1× PBS for 1 hour and stained with primary antibodies diluted in blocking solution for 48 hours. After three washes with 1% BSA/0.15% saponin in 1× PBS, we stained the organoids with secondary antibodies for 24 hours, stained them with Hoechst for 1 hour, washed twice, and mounted them on the glass slide with Immu-Mount. We used the following antibodies: mouse anti-Map2 (clone AP-20, Sigma-Aldrich), mouse anti–β-III-tubulin (clone SDL.3D10, Sigma-Aldrich), rabbit anti-GFAP (polyclonal, Dako), rabbit anti-Nestin (polyclonal, Sigma-Aldrich), Alexa-Fluor 488 goat anti-mouse, and Alexa-Flour 568 goat anti-rabbit immunoglobulin G.

### RNA extraction and RT-PCR

We extracted RNA from the NPCs and brain organoids at 2, 4, 8, and 10 weeks of differentiation using the Quick RNA extraction kit (Zymo). We quantified the integrity of RNA with NanoDrop. We reverse-transcribed cDNA using M-MLV Reverse Transcriptase (Promega) and random hexamer primers as described previously (*44*). We used the TaqMan gene expression assay to perform real-time polymerase chain reaction (RT-PCR).

### Encapsulation of brain organoids in the 3D shell electrodes

As the shell electrodes folded up, we sterilized the glass chamber and 3D shell electrodes with 70% ethanol. We rinsed the device with 1× PBS and then cell medium. Later, we added the organoid into the chamber with a pipette and used a pipette tip to gently move the organoid into the 3D shell electrodes from the non-leaflet direction.

### Calcium imaging

For calcium imaging, we first fabricated the 3D shell electrodes on a transparent quartz wafer. After organoid encapsulation, we applied 0.4× fluo-4 direct calcium reagent (Sigma-Aldrich, St. Louis, MO) into the organoid medium and incubated it at 37°C for 1 hour and then let it rest at room temperature for 15 min before imaging. We imaged the sample with a Nikon A1 confocal microscope (Nikon, Tokyo, Japan).

### Cytotoxicity test

We used a LIVE/DEAD viability/cytotoxicity kit for mammalian cell (Molecular Probes, Eugene, OR). We added 20 μl of 1 mM calcein AM and 20 μl of 2 mM ethidium homodimer-1 in 10 ml of PBS solution. We added the chemicals to brain organoids for 10 min and then rinsed with PBS and medium before imaging with a Nikon AZ100 microscope (Nikon, Tokyo, Japan).

### SEM imaging

We sputter-coated gold on the 3D shell electrodes for 1 min. We took scanning electron microscopy (SEM) images using a JEOL SEM (JSM IT100).

### FEM simulation

We used FEM with Abaqus to simulate the folding of SU8 bilayers. We modeled each layer of the SU8 bilayer and the gold electrode as homogeneous, isotropic material. The mechanical properties of each layer of the SU8 bilayer were defined by its Young’s modulus (*E*), Poisson’s ratio (=0.3), and preexisting strain (ɛ) before folding. Additional details of the model are in the Supplementary Materials.

### 2D recording and 3D recording process

For the 2D recordings with flat (prefolded) SU8 bilayer shells, we first coated the 2D MEAs with Matrigel solution and incubated it for 1 hour. We then replaced the Matrigel solution with cell medium and placed the brain organoid on the electrodes. Before starting the recording, we put the device and the organoid back into the incubator for approximately 24 hours for neurite outgrowth and environment stabilization. For 2D recordings with the commercial Axion system, the MEA plate was coated with poly-l-ornithine (15 μg/ml) and laminin (10 μg/ml).

For the 3D recordings, after actuation and sterilization of the shell electrodes, we loaded the cell medium and placed the organoid onto the semifolded shell electrodes. The shell continued to fold to firmly encapsulate the brain organoid. We put the device and the organoid back into the incubator for 24 hours or a longer time for environment stabilization before we started the recording. No protein or gel coatings were used for 3D shell MEA recordings.

### Recording data acquisition

For the electrophysiology recording, we connected the outputs of the shell electrodes to the printed circuit board (PCB) interface using micro alligator clips. We connected the Omnetics connector on the PCB to a 32-channel headstage, transferring the electrophysiology recording to an RHD recording controller. The sampling rate of the recording was 20 kHz. We acquired all recordings in a grounded Faraday cage on a vibration isolation table.

### Signal processing

After preprocessing the raw signals, we detected spikes separate for each channel using a threshold-based method, where the threshold is automatically set to be 5σ*_n_*. We estimated the noise level σ robustly using the formula σ*_n_* = median(∣*S_t_*∣/0.6745), where ∣*S_t_*∣ is the absolute signal amplitudes. After spike locations were detected, we isolated them by windows of length 3.5 ms. We then regarded locations of the largest amplitudes as event times.

### Spike merging

For the comparison of 3D and 2D channels, we merged the spikes so that one spike train represented the 3D recordings and one represented the 2D recordings. To accomplish this, we detected and merged identical spikes by multiple electrodes to avoid double counts. This was done by treating spikes with peak times differing less than 2 ms (one waveform length) as the same spike and, hence, summarizing them as one single spike with maximum normalized amplitude (*45*, *46*).

### Spike comparison between 3D and 2D recordings

After merging spikes into one sequence for 3D electrodes and one sequence for 2D electrodes, we detected 7785 spikes from the 3D electrodes and 2025 spikes from 2D electrodes; among these, 1860 spikes were detected in common by both electrodes. We calculated the normalized spike amplitudes by dividing the spike amplitudes by noise level, σ*_n_* (henceforth SNR).

We also compared the sensitivity of 3D and 2D recording in response to stimulation. We stimulated the organoid with glutamate, yielding five rounds of recording data. Since glutamate is known to increase neural activity (*37*), we investigated whether 3D or 2D electrodes are more sensitive to glutamate-mediated changes. We quantified the trend via the Mann-Kendall’s *z* statistics of spike amplitudes (*38*). This statistic is larger for increasing trends and is normalized to be comparable across different recordings. We used the permutation test, permuting the 2D versus 3D labels, for statistical inference.

## Supplementary Materials

### This PDF file includes:

Figs. S1 to S17 Tables S1 and S2 Notes S1 and S2 References
