# Supplementary material for: Shell microelectrode arrays (MEAs) for brain organoids
Source: Sci Adv. 2022 Aug 17;8(33):eabq5031. doi: 10.1126/sciadv.abq5031 (PMC9385157; doi:10.1126/sciadv.abq5031)
Supplement: Supplementary file 1 — Figs. S1 to S17 Tables S1 and S2 Notes S1 and S2 References [file sciadv.abq5031_sm.pdf]

Science Advances
AAAS

Supplementary Materials for

Shell microelectrode arrays (MEAs) for brain organoids

Qi Huang *et al.*

Corresponding author: David H. Gracias, dgracias@jhu.edu

Sci. Adv. **8**, eabq5031 (2022)
DOI: 10.1126/sciadv.abq5031

This PDF file includes:

Figs. S1 to S17
Tables S1 and S2
Notes S1 and S2
References

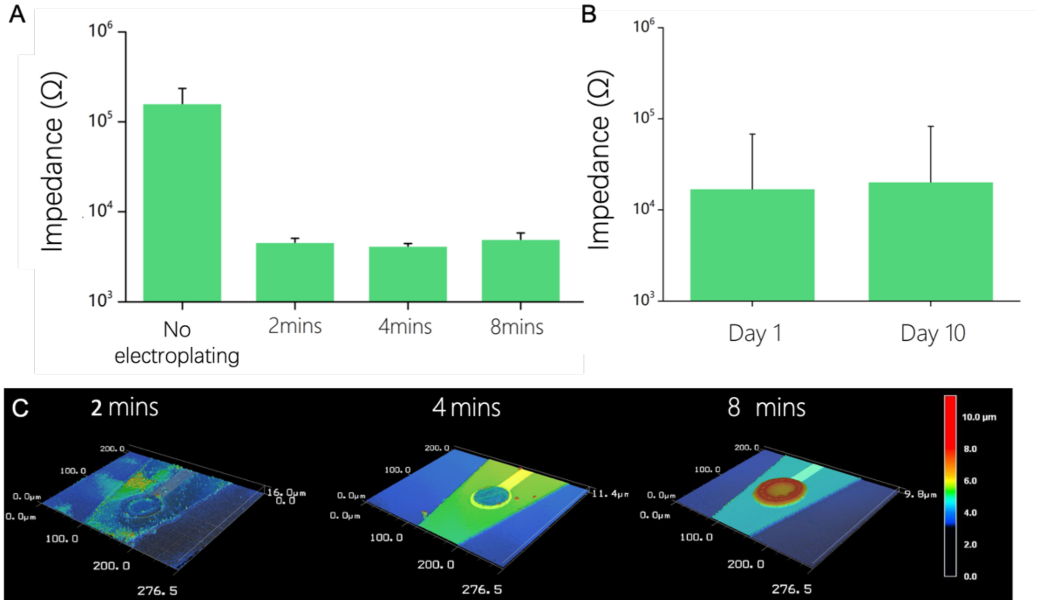

Fig. S1. Electrodeposition characteristics of PEDOT: PSS

A) Plot showing the electrode impedance as a function of the conductive polymer PEDOT: PSS electroplating time. (B) Plot showing the impedance change of 6 PEDOT: PSS electrodes after 10 days of soaking in cell medium. The data indicates that the impedance does not change significantly over this time. (C) Laser scanning microscopy height profiles of the PEDOT: PSS coated gold electrodes at different electroplating times.

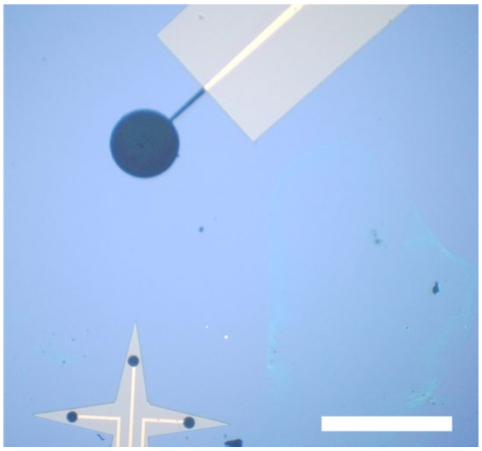

Fig. S2. Location of the reference electrode relative to the shell MEA.
Optical image showing the position of the reference electrode relative to the shell electrodes in the pre-folded state. Scale bar: 1000 μm.

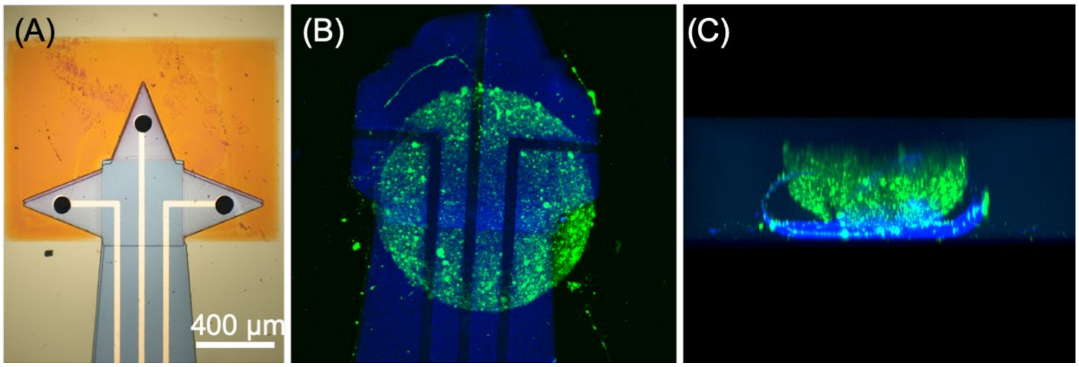

Fig. S3. Initial electrode configuration and brain organoid encapsulation.

(A) Image of a 3D shell MEA in the pre-folded (as-fabricated) state. (B) Top view of a fluorescently labelled brain organoid captured within a 3D shell MEA. (Blue: SU8, Green: Fluo-4 labeled brain organoid) (C) Side view showing an example of self-folded leaflets not able to accurately encapsulate the brain organoid suggesting the need for optimization of leaflets and folding.

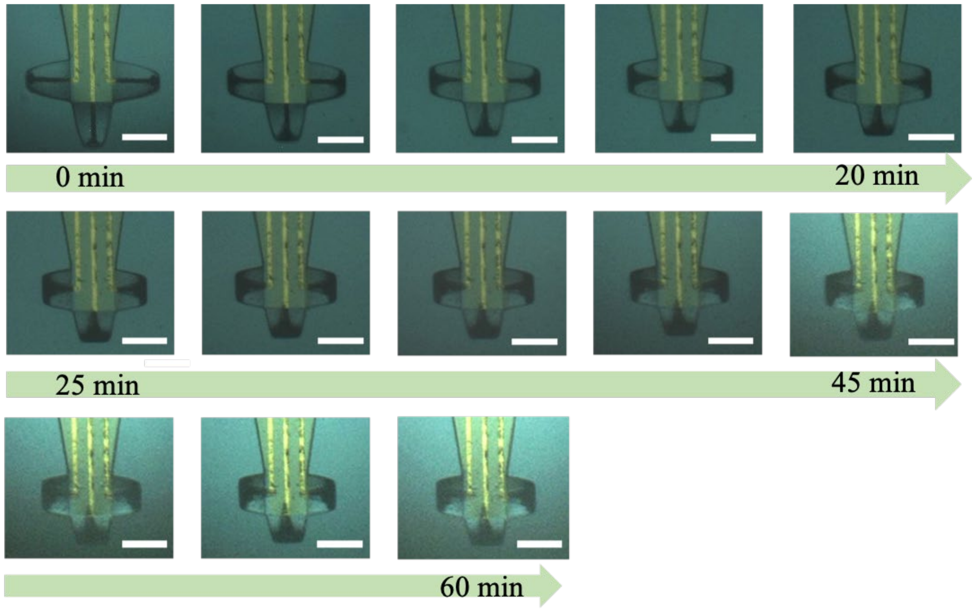

Fig. S4. Typical time scale of the self-folding of the 3D shell MEAs

Time-lapse images showing the self-folding process of the 3D shell MEAs. The folding process is slow enough to enable secure placement and capture of the organoid. Scale bar: 250 µm.

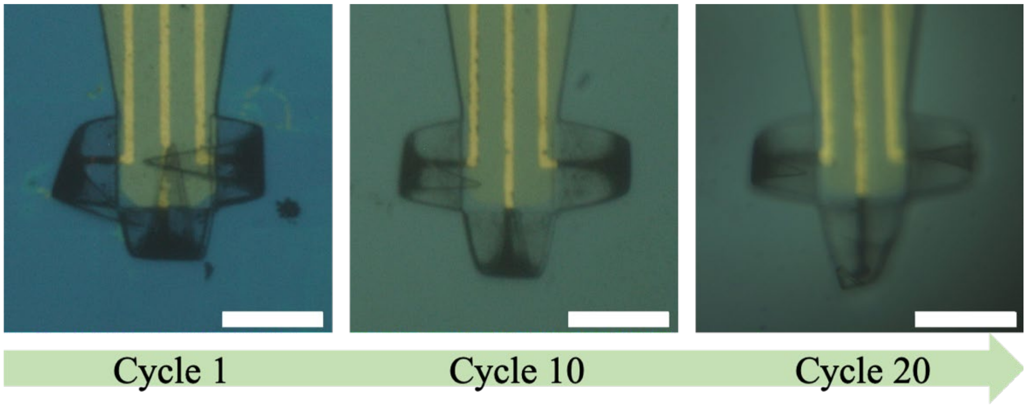

Fig. S5. Reversibility of self-folding of the shell MEAs

Image snapshots showing the reversible folding of the 3D shell electrodes. In a single cycle, we put the 3D shell MEA back into acetone from water to flatten it, then we put it back into water to fold it up. This reversible folding and unfolding offers the potential for reuse of the MEAs. Scale bar: 250 μm.

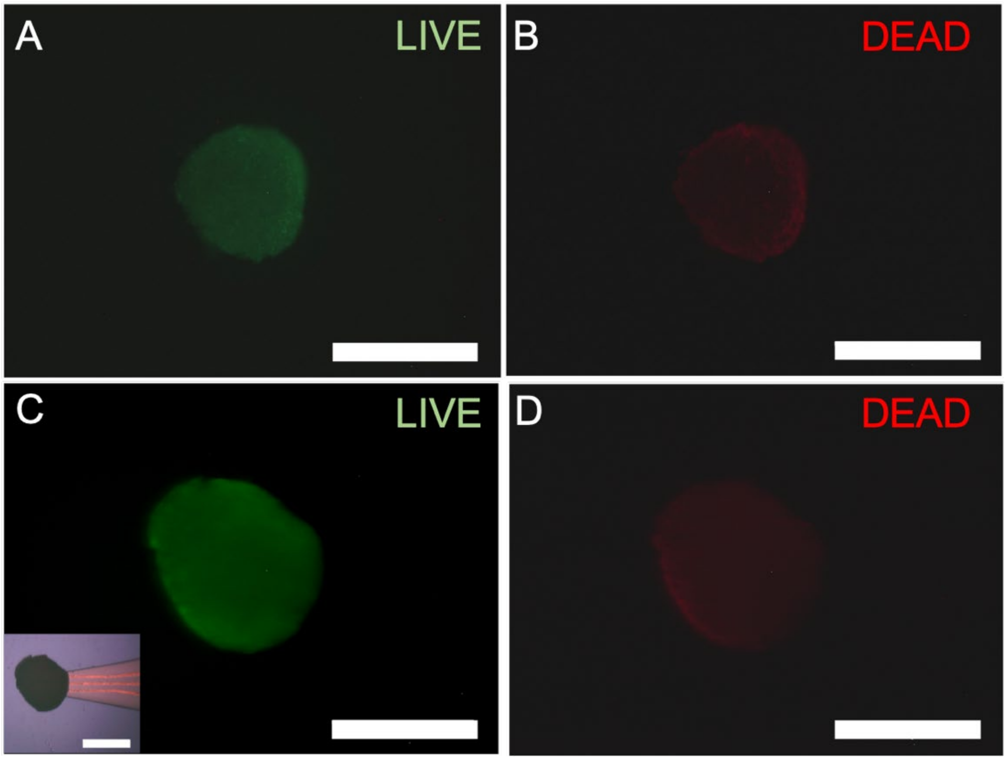

Fig. S6. Biocompatibility of the shell MEA.

Live/dead assay of (A-B) free-floating and (C-D) brain organoid encapsulated in a shell MEA after 24 hours, the free-floating brain organoid A) Calcein AM (green; live); B) Ethidium homodimer-1 (red; dead); the brain organoid encapsulated C) Calcein AM (green; live); within shell MEA, D) Ethidium homodimer-1 (red; dead); within shell MEA. Results indicate biocompatibility of the shell MEA. Scale bar: 500 μm.

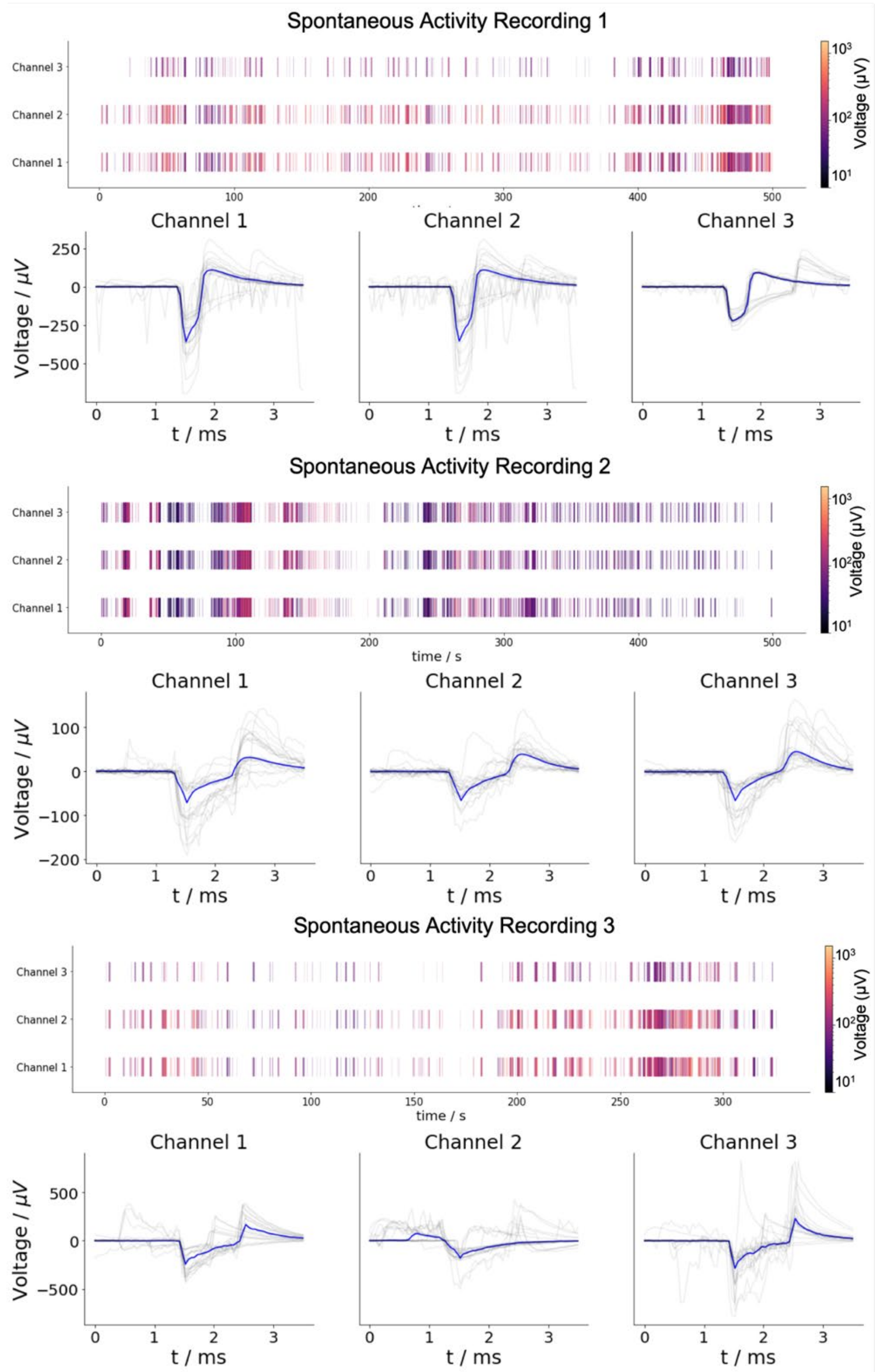

Fig. S7. Recordings of spontaneous activities of brain organoids measured by the 3D shell MEAs.

The three recordings are from three different organoids.

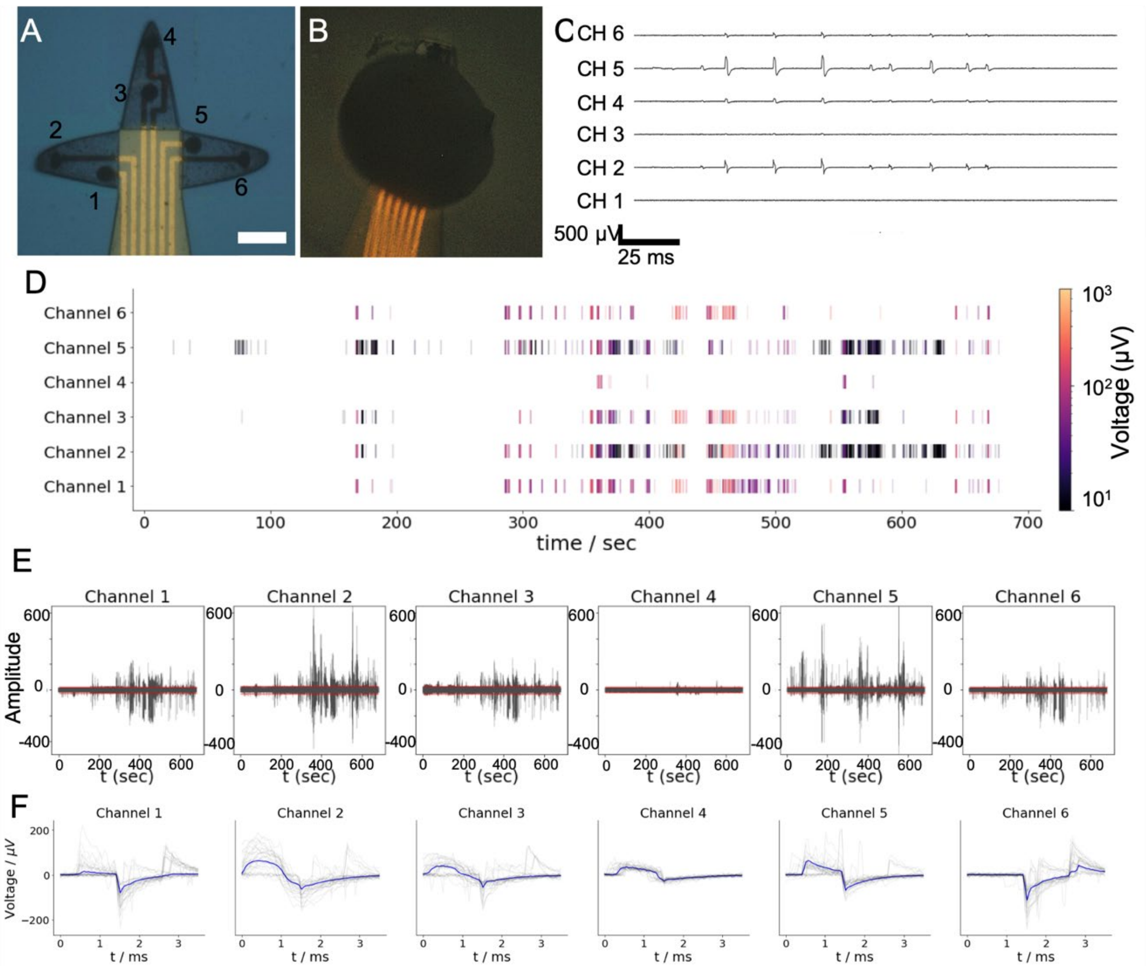

Fig. S8. 3D shell MEA recordings from brain organoids in 6-channel (two on each leaflet) shell electrodes.

(A) Optical image of the 6-channel 3D shell MEAs. Scale bar: 200 µm. (B) Optical image of a 6-channel 3D shell MEAs encapsulating a brain organoid. (C) Field potential recorded from the 6-channel 3D shell MEAs. (D) Representative raster plot of the recording. (E) Field potential recorded from 6-channel 3D shell MEAs over approximately 10 minutes. (F) Overlaid spike waveform of each channel.

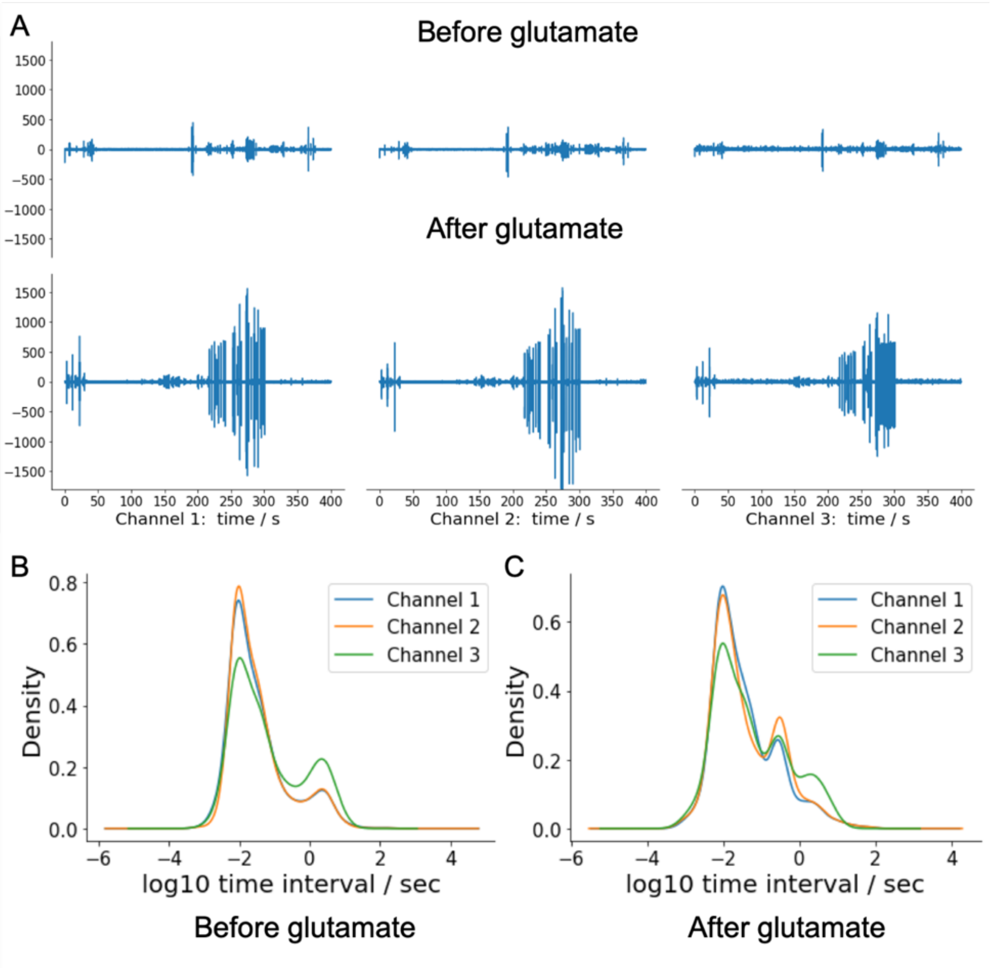

Fig. S9. 3D MEA shell recordings with glutamate stimulation

(A) 3D MEA shell recordings from a brain organoid before and after glutamate stimulation. (B-C) Inter-spike-interval (ISI) density change (B) before and (C) after glutamate stimulation. The new crest emerged around -0.5 indicating the glutamate-related spikes had a longer ISI.

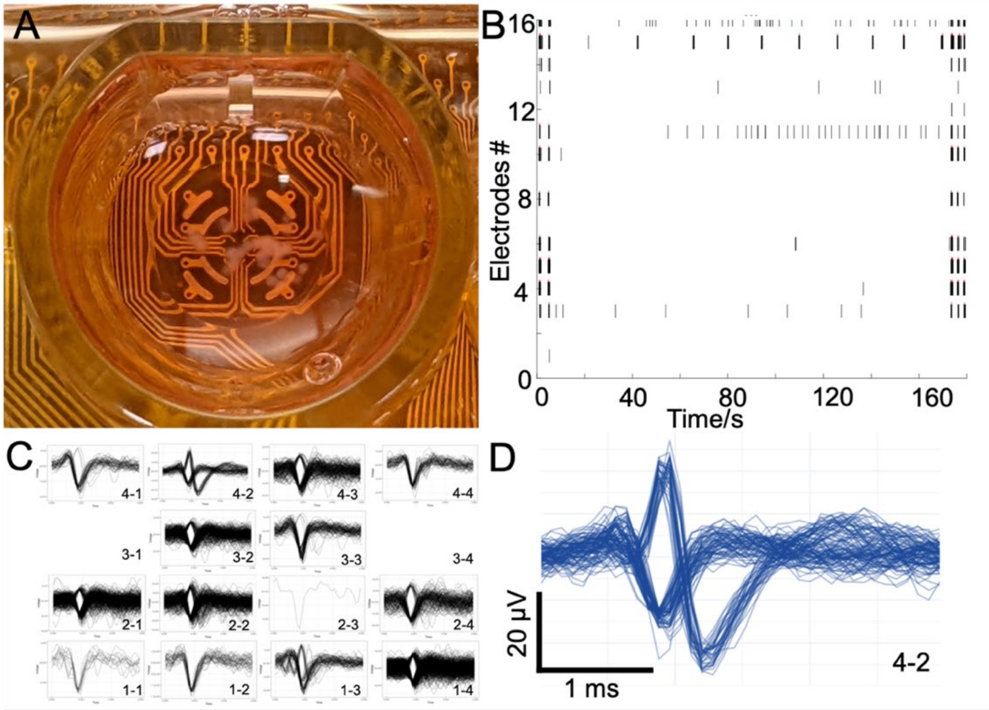

Fig. S10. Brain organoid recordings using a conventional 2D Maestro MEA system (Axion Biosystem, Atlanta).

(A) An optical image of organoids on a commercial MEA plate. The diameter of the well is 10.35 mm. (B) Raster plot of the recording from the MEA plate. (C) Spike waveform of 16 channels from the recording from 2D MEA plate. (D) Zoomed-in spike waveform of a representative channel (4-2).

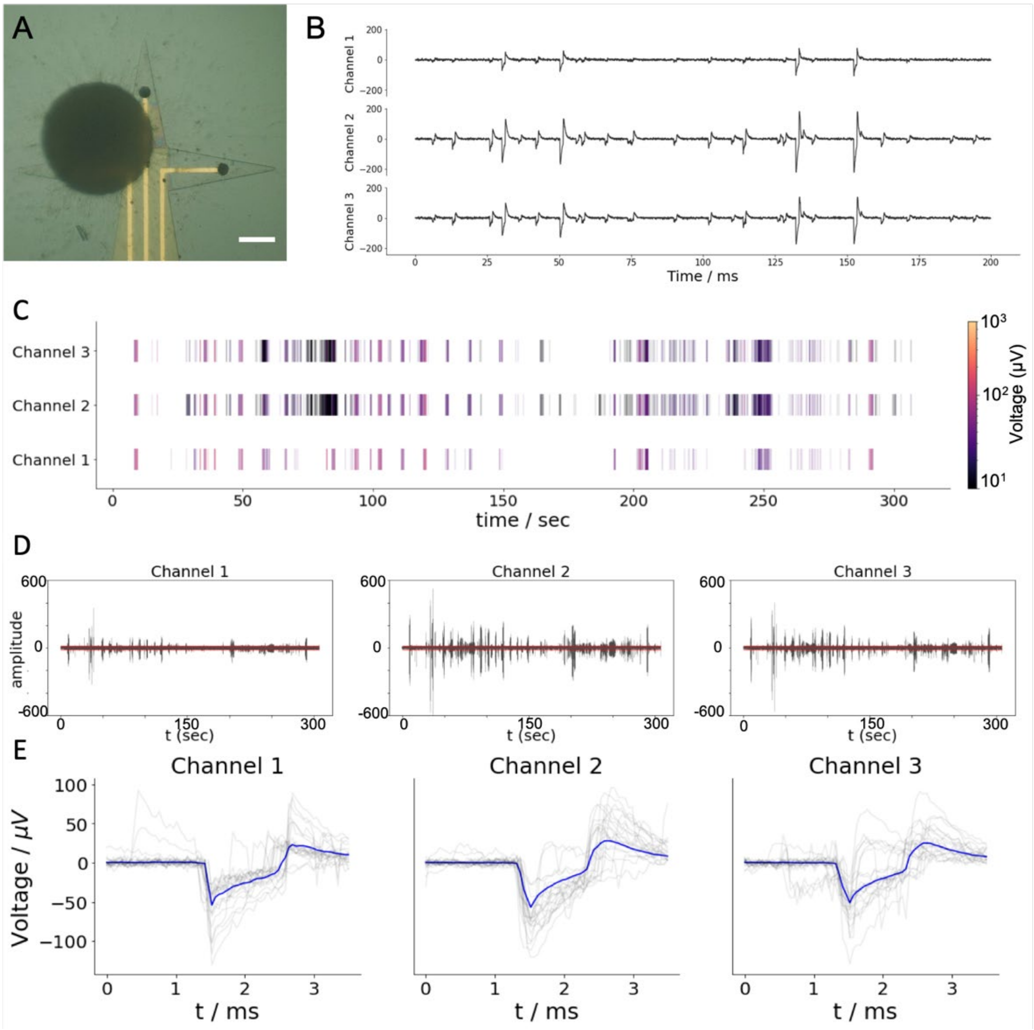

Fig. S11. 2D recording with shell electrodes in pre-folded state.

(A) Optical image of the brain organoid placed on the electrodes. Scale bar: 200 µm. (B) Field potential recorded from 2D electrodes. (C) Representative raster plot of the recording. (D) Field potential recorded from 2D electrodes on a larger time scale. (F) Overlaid spike waveform of each channel.

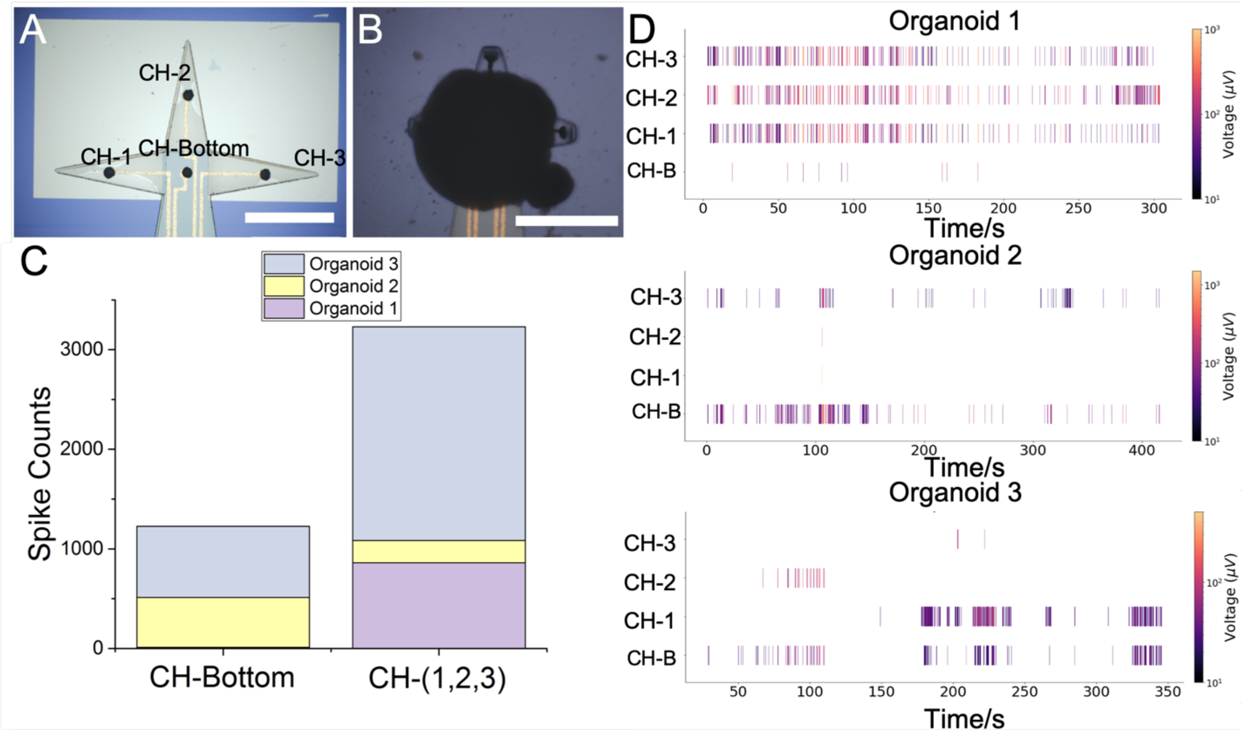

Fig. S12. Comparison of recordings from 2D and 3D electrodes within a shell MEA.

(A) Shell MEAs with three electrodes on the leaflets and an additional electrode at the bottom center. (B) Image of an encapsulated organoid within a shell MEA. (C) Stacked spike counts detected from only the bottom electrode as compared to the shell electrodes indicating that that the union of spike events from all over the shell (3D) is greater than that from a single planar 2D electrode. (D) Detailed raster plot of the recordings from the four different electrodes. Scale bar: 500 µm.

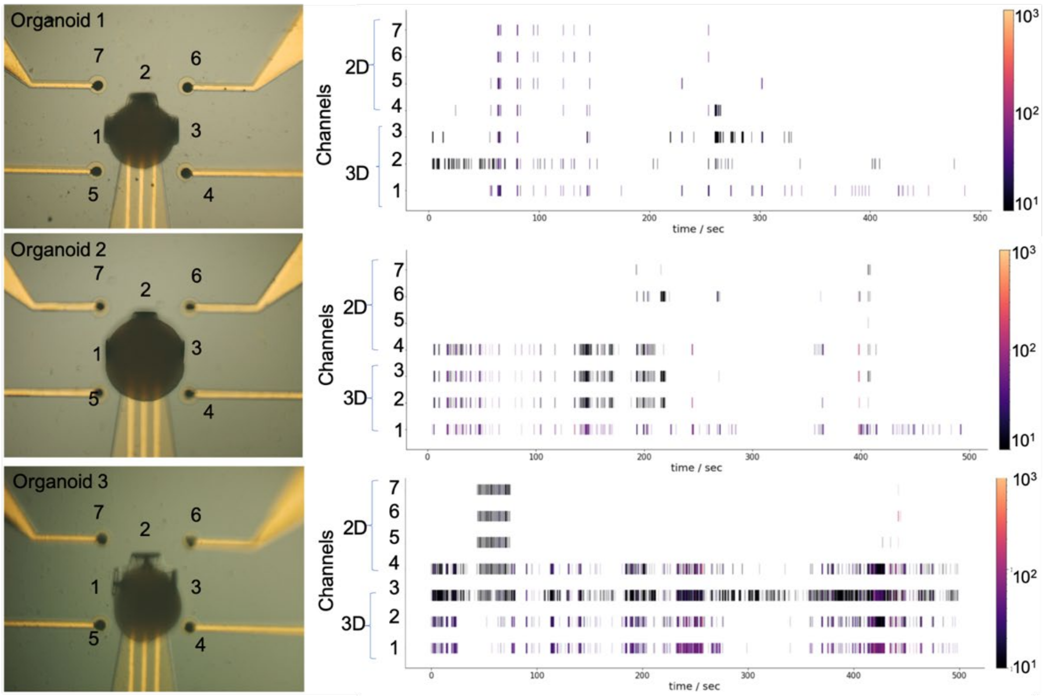

Fig. S13. Recording of the spontaneous activities from brain organoids recorded using both 2D and 3D shell electrodes at different distances. No protein or gel coatings were used.

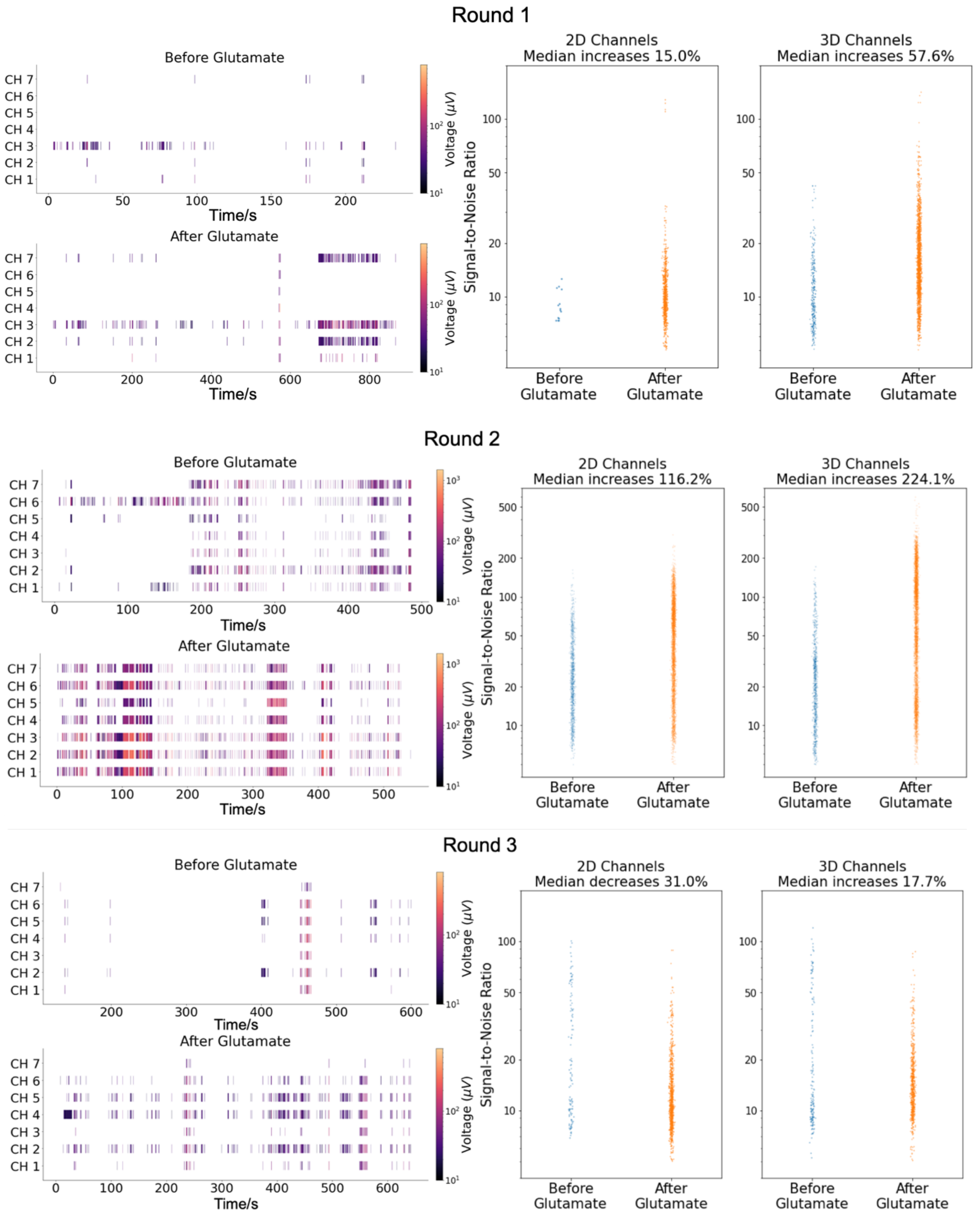

Round 1
Before Glutamate
After Glutamate
CH 7
CH 6
CH 5
CH 4
CH 3
CH 2
CH 1
Time/s
Voltage (μV)
2D Channels
Median increases 15.0%
3D Channels
Median increases 57.6%
Signal-to-Noise Ratio
Before Glutamate
After Glutamate
Round 2
2D Channels
Median increases 116.2%
3D Channels
Median increases 224.1%
Round 3
2D Channels
Median decreases 31.0%
3D Channels
Median increases 17.7%

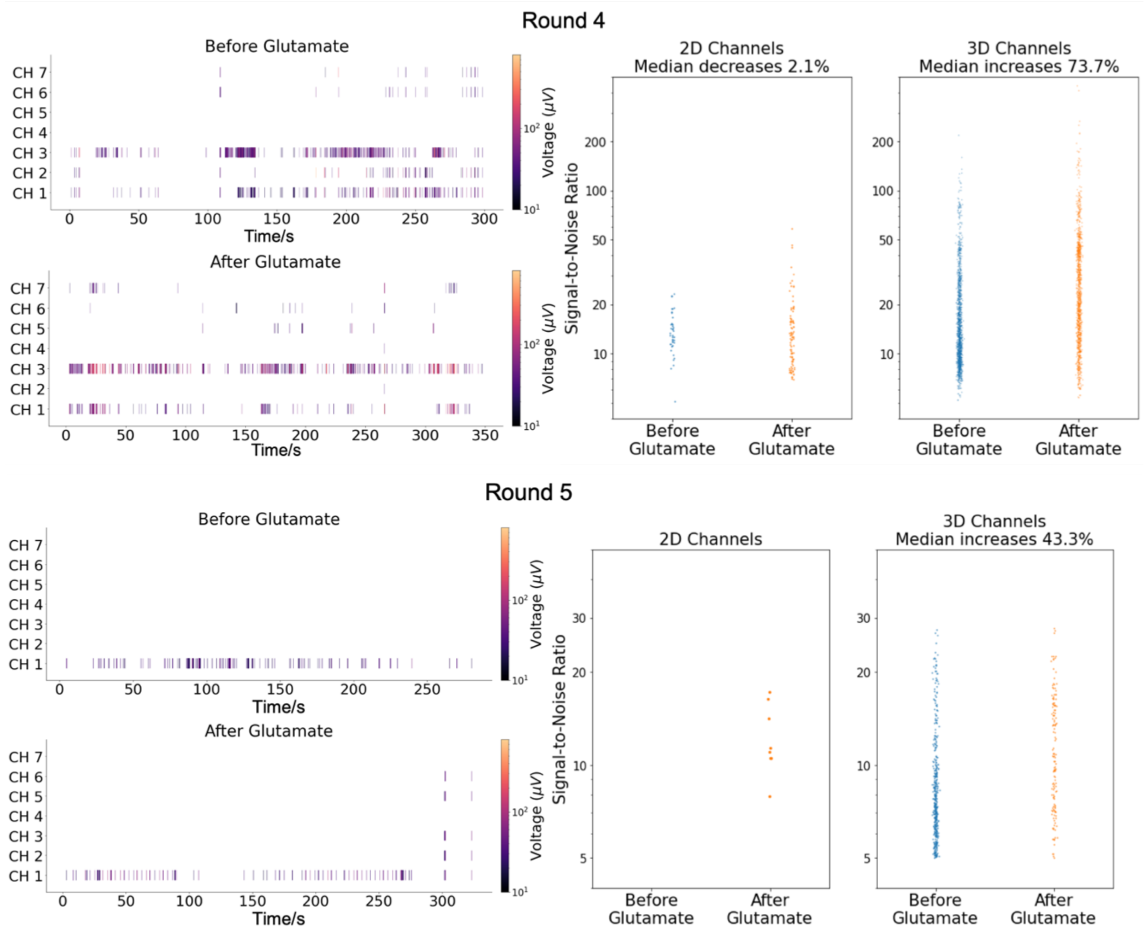

Fig. S14. Brain organoid recordings over five rounds of glutamate using both 2D and 3D shell electrodes. No protein or gel coatings were used.

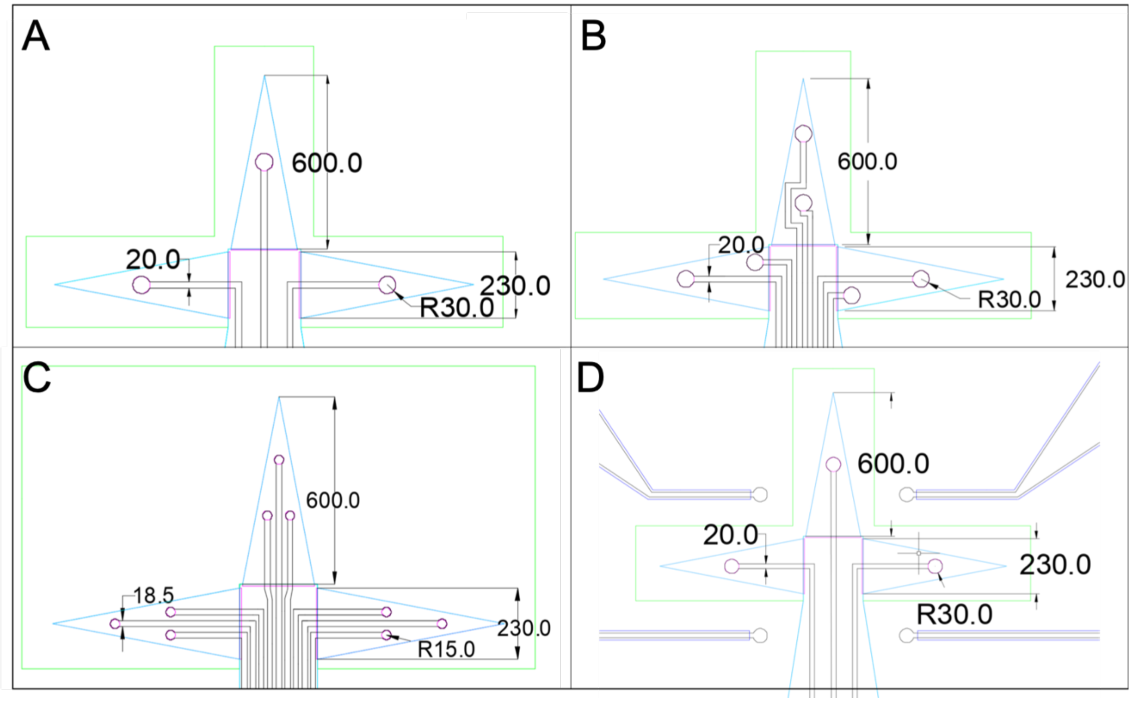

Fig. S15. CAD mask drawings showing the dimensions of the shell electrodes.
Design with (A) three electrodes, (B) six electrodes, (C) nine electrodes, (D) 2D vs 3D. The numbers shown are in micrometers.

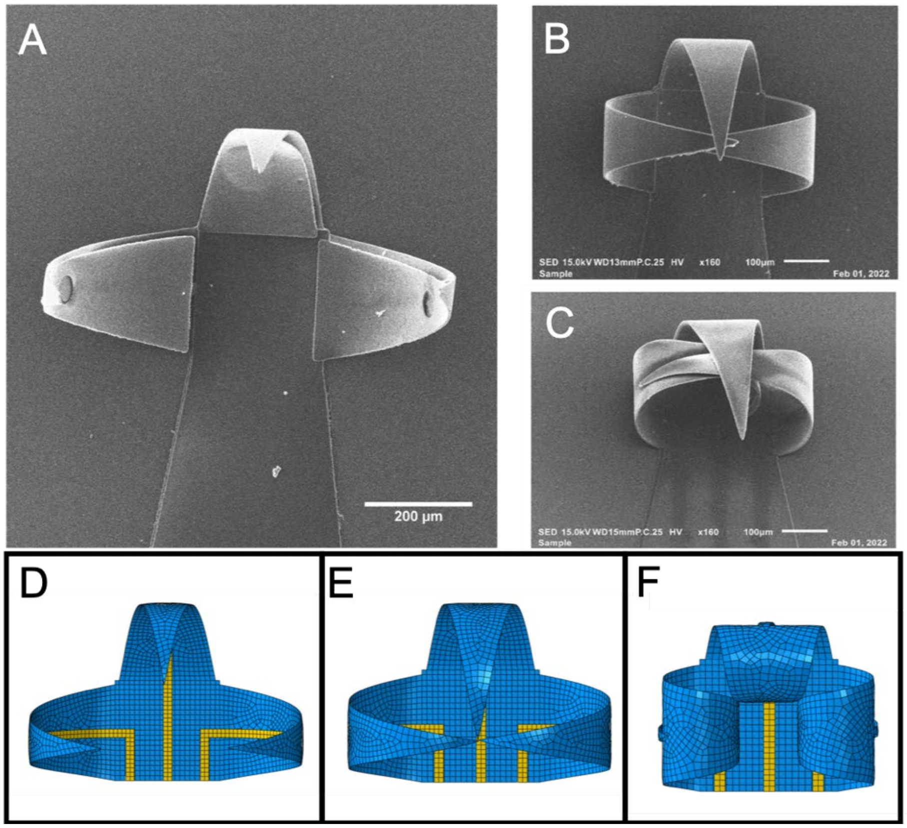

<

Fig. S16. Comparison between experiments results and mechanics simulations for self-folding of the shell MEAs.

Comparison between images of experimentally fabricated self-folding SU8 leaflets (top) and FEM simulations (bottom row). Here we show polymers of different thicknesses and top layer exposures have similar curvature shapes. Images (A) and (D) are 8μm (total thickness) SU8 exposed at 120 mJ/cm^2. (B) and (E) are 6μm (total thickness) exposed at 180 mJ/cm^2, and images (C) and (F) are 4.6μm exposed at 180 mJ/cm^2. As the curvature increases, the leaflet tips begin to rest on each other. While our FEM models do not capture this polymer interaction at the leaflet tips, the radius of curvature measurements support the degree of curvature in the laboratory models. Our experiment also assumes that the presence of organoids within the device will prevent full curving of the leaflets, as shown on Fig. 2B.

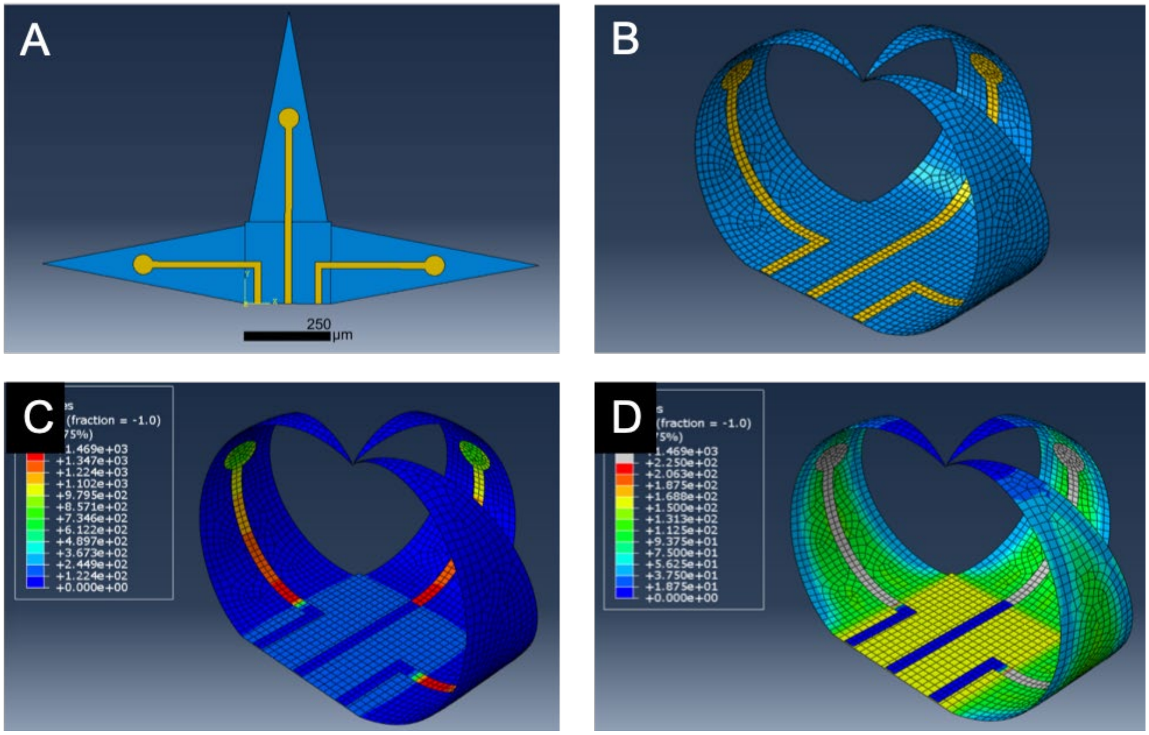

Fig. S17. Abaqus self-folding modeling represenatation for shell MEAs.

Abaqus model representation of a 6 μm thick shell whose top layer is exposed to 180 mJ/cm^2 UV light. (A) Top view with the gold electrodes in yellow, and SU8 polymer shown in blue. (B) Image showing the fully deformed shape after 43 steps. (C) Image showing stress distribution in the overall MEA shell and shows that gold electrodes experience the highest stress. (D) Image representing the stress distributions in the SU8 material alone, showing high stresses at the bottom surface.

Table S1. Modeling parameters used

The Young's modulus as a function of the energy of exposure amount. The data was experimentally measured.

Exposure energy *I* (mJ/cm^2)	Young's modulus *E* ± STDEV (GPa): ***Experiment***	Young's modulus *E* ± STDEV (GPa): ***FEM*** as given by Eq. 1
56.75	0	0.0129
120	4.99±0.38	4.99
196	5.95±0.04	5.99
240	6.13±0.07	6.10

Table S2. Modeling parameters used

The temperature gradient as a function of bilayer shell thickness and varying top layer energy exposures, and average radius of curvature of the folded leaflets.

Total thickness of SU8 bilayer (μm)	Top layer UV exposure I_{Top}(mJ/cm^2)	Temperature gradient D_{eps}	Average radius of curvature (μm)
4.6	120	-25.81	47.33
	150	-17.28	46.57
	180	-13.62	54.40
6.0	120	-11.63	70.80
	150	-7.79	108.02
	180	-6.14	135.16
8.0	120	-4.91	177.70
	150	-3.29	274.59
	180	-2.59	328.53

Supplemental Note S1: Mechanical modeling of SU8 in different cross-linking states

We used the finite element method (FEM) to simulate the self-folding of the SU8 bilayers. We modeled each layer of the SU8 leaflet and the gold electrode as a homogenous, isotropic material. The mechanical properties of each layer of the SU8 bilayer are defined by its Young's modulus (E), Poisson's ratio (ν=0.3) and pre-existing strain (ε) before folding.

To model the Young's modulus of SU8 as a function of the UV exposure, we set up a series of experiments to measure the material stiffness as a function of the exposure energy for a 5 µm film. Each energy level was repeated nine times, and we measured the Young's modulus of the film after curing. Since it is challenging to measure the critical energy level that starts to cure the material, we considered 56.75 mJ/cm^2, as the critical energy obtained from the previous analysis, as the energy level to transition SU8 from a liquid to solid state and thus corresponds to zero Young's modulus, as summarized in **Table S1** which gives the experimental value of Young's modulus of the SU8 film after the specified energy amount of UV exposure.

By using the values in table S1, we obtained the exponential decay function for the sample stiffness as a function of the exposure energy. We identified the Young's modulus as a function of exposure energy as,

$$E(k) = E_0 - \Delta E \cdot exp(-k/k_0) \qquad (1)$$

where $E_0 = 6.15$ GPa is the limiting Young's modulus with enough exposure, $\Delta E = 27.26$ GPa and $k_0 = 38.06$ mJ/cm^2 are two constants obtained through fitting and determine the shape of the curve. Eq. (1) provides the basic function for the Young's modulus of the top/bottom SU8 layers in the experiments.

We considered the effect of the solvent exchange on the top/bottom SU8 layers by tuning the pre-existing strain ε in each layer of the bilayer to reflect the volume change after being rinsed in acetone and put into water. Several reports in the molecular dynamics (MD) literature have discussed the volume/density of epoxy-based polymer materials after crosslinking. However, there are contradicting results as some show that a higher crosslink ratio causes the material to shrink while others show that the material swells. However, these volume changes are on the order of only a few percent [47-48] and are not large enough to explain the large-scale deformation of the experimental samples in our current study. Here, we considered that the volume change that happens to the top/bottom layers is caused by dissolving of uncrosslinked SU8 monomers in acetone, leaving voids that cause the material to shrink in water because of the highly hydrophobic features of SU8. According to the theory of rubber-like elasticity, the crosslink density n can be calculated from the Young's modulus E and the absolute temperature T as,[49]

$$n = \frac{E}{3RT} \qquad (2)$$

where R is the gas constant. It is clear that $n \propto E$ and thus we have the ratio of crosslinked SU8 monomers given by,

$$c = \frac{n}{n_0} = \frac{E(k)}{E_0} \qquad (3)$$

where n_0 is the fully crosslinked SU8 corresponding to a Young's modulus of E_0. Based on the above analysis, the final volume V_{end} of the material after dissolving in acetone and shrinkage in water is given by the initial volume V_0 as $V_{end} = AcV_0$, where A is an unknown factor and $A = 1$ simply means all uncrosslinked SU8 gets dissolved in acetone. Hence, the pre-existing strain of the equilibrium SU8 bilayer before folding, in all the directions, is given by,

$$\varepsilon = \sqrt[3]{Ac} - 1, \tag{4}$$

We note that there is only one unknown parameter A for our material model given by Eqs. (1) and (4). We estimated its value by testing different *A's* and comparing them to the experimental results for a specific leaflet curvature. We identified A=0.87 as yielding a radius of curvature that gives the closest results when compared to our experimentally obtained models. All the simulation results reported in the main text are obtained by using this constant value.

Supplemental Note S2: Finite element modeling of the SU8 bilayer under thermal strains

We developed a FEM model in Abaqus to predict the 3D folded structure of a bilayer SU8 leaflet from a 2D pattern design. We modeled the geometry of the bilayer leaflets (2D pattern, thickness), the Young's modulus, Poisson's ratio and pre-existing strain with Abaqus CAE and solved for the fully equilibrated folded structure by using the Standard/Implicit solver on our local workstation (**Table S2**). To make the simulation efficient, we modeled the initial pattern of the SU8 bilayers by a 3D planar shell with the thickness defined by the actual polymer bilayer (t) and enough integration points (11) along the thickness to account for the heterogeneous normal strain distribution, which provides the driving force for folding. To apply the mismatch strain to the top and bottom layer (as given by the different pre-strain ε_{top} and ε_{bottom}), we prescribed a predefined temperature field by directly assigning the temperature (T) gradient, $\frac{dT}{dt}$ through the bilayer thickness, which is given by,

$$\frac{dT}{dt} = \frac{\varepsilon_{top}}{C_1 \alpha t^{C_2}} \tag{5}$$

where ε_{top} gives the mismatch strain of the top and bottom layer (we assume the strain in the bottom layer is zero as it is always fully cross-linked by exposing to UV of = 240 mJ/cm^2) in the FEM bilayer model as given Eq. (4), α can be any positive value for the thermal expansion coefficient as defined for the material property (we use 0.001 /ºC here), and t is the thickness of the bilayer. We included two numerical parameters C_1 and C_2 here, because the mismatch strain occurs only at the interface between two bonded layers in experiment, instead of a continuous gradience of strain as what is used in FEM. We observed in experiments that the conformation of the 3D folded structure was much more sensitive to the change of t of a small value than t of a large value. We systematically varied the C_1 and C_2 values and compared the simulations with the experimental observations for bilayers with different thicknesses and UV to identify that $C_1 = 0.35$ and $C_2 = 3$ yield the best match (**Figure S16**).

We modeled the gold (Au) electrode layer by another shell with a thickness of 85 nm and material properties given by (E=79 GPa, ν=0.415). We assembled this layer on top of the SU8 bilayer surface, as shown in **Figure S17**.

We also rationalized the relationship between the radius of curvature as a function of UV exposure intensity by post-processing the deformed Abaqus/Standard models. We performed further processing of the deformed Abaqus/Standard models in the computer-aided-design (CAD) software SOLIDWORKS, where we visualized the radius of curvature of the leaflets. Our analysis showed a positive correlation between the radius of curvature, shell thickness, and top layer UV exposure amount.

We exported a raw object mesh file (OBJ) consisting of around 6,000 triangles from Abaqus into the SOLIDWORKS software for this analysis. OBJ is a simple file format that represents the 3D geometry alone. We chose SOLIDWORKS to analyze the model due to the ease and availability of tools that can accurately describe the radius of curvature along different parts of a curved surface. To capture the radius of curvature, we identified a set of points along the centerline of any of the three leaflets. In our simulations, we assumed all three folded leaflets had the same curvature and fold shape. We chose the slicing tool along on a geometric plane parallel to the curved leaflet to generate a two-dimensional sketch of points. We converted the approximately 130 points formed using this "Slice" into a spline line, with a tolerance matching the thickness of each model. It is important to note that the curvature along the leaflets is not a perfect circle but

elliptical in shape. To accurately describe the radius of curvature, we examined the bottom, middle, and top thirds of each leaflet, where the SOLIDWORKS's spline line feature was able to generate a minimum radius curvature of a circle that could fit the chosen set of points.

In total, we measured four radii at the bottom, middle, top, and whole leaflet, then averaged these together to generate a numerical description of the degree of folding.

REFERENCES AND NOTES

1. T. Hartung, Thoughts on limitations of animal models. *Parkinsonism Relat. Disord.* **14**, S81–S83 (2008).

2. E. Di Lullo, A. R. Kriegstein, The use of brain organoids to investigate neural development and disease. *Nat. Rev. Neurosci.* **18**, 573–584 (2017).

3. C. A. Trujillo, R. Gao, P. D. Negraes, J. Gu, J. Buchanan, S. Preissl, A. Wang, W. Wu, G. G. Haddad, I. A. Chaim, A. Domissy, M. Vandenberghe, A. Devor, G. W. Yeo, B. Voytek, A. R. Muotri, Complex oscillatory waves emerging from cortical organoids model early human brain network development. *Cell Stem Cell* **25**, 558–569.e7 (2019).

4. U. Marx, T. Akabane, T. B. Andersson, E. Baker, M. Beilmann, S. Beken, S. Brendler-Schwaab, M. Cirit, R. David, E. M. Dehne, I. Durieux, L. Ewart, S. C. Fitzpatrick, O. Frey, F. Fuchs, L. G. Griffith, G. A. Hamilton, T. Hartung, J. Hoeng, H. Hogberg, D. J. Hughes, D. E. Ingber, A. Iskandar, T. Kanamori, H. Kojima, J. Kuehnl, M. Leist, B. Li, P. Loskill, D. L. Mendrick, T. Neumann, G. Pallocca, I. Rusyn, L. Smirnova, T. Steger-Hartmann, D. A. Tagle, A. Tonevitsky, S. Tsyb, M. Trapecar, B. van de Water, J. van den Eijnden-van Raaij, P. Vulto, K. Watanabe, A. Wolf, X. Zhou A. Roth, Biology-inspired microphysiological systems to advance patient benefit and animal welfare in drug development. *ALTEX* **37**, 365–394. (2020).

5. A. Roth, Human microphysiological systems for drug development. *Science* **373**, 1304–1306 (2021).

6. M. A. Lancaster, M. Renner, C.-A. Martin, D. Wenzel, L. S. Bicknell, M. E. Hurles, T. Homfray, J. M. Penninger, A. P. Jackson, J. A. Knoblich, Cerebral organoids model human brain development and microcephaly. *Nature* **501**, 373–379 (2013).

7. A. M. Paşca, S. A. Sloan, L. E. Clarke, Y. Tian, C. D. Makinson, N. Huber, C. H. Kim, J.-Y. Park, N. A. O'Rourke, K. D. Nguyen, S. J. Smith, J. R. Huguenard, D. H. Geschwind, B. A. Barres, S. P. Paşca, Functional cortical neurons and astrocytes from human pluripotent stem cells in 3D culture. *Nat. Methods* **12**, 671–678 (2015).

8. D. Pamies, P. Barreras, K. Block, G. Makri, A. Kumar, D. Wiersma, L. Smirnova, C. Zang, J. Bressler, K. M. Christian, G. Harris, G.-L. Ming, C. J. Berlinicke, K. Kyro, H. Song, C. A. Pardo, T. Hartung, H. T. Hogberg, A human brain microphysiological system derived from induced pluripotent stem cells to study neurological diseases and toxicity. *ALTEX* **34**, 362–376 (2017).

9. B. Cakir, Y. Xiang, Y. Tanaka, M. H. Kural, M. Parent, Y.-J. Kang, K. Chapeton, B. Patterson, Y. Yuan, C.-S. He, M. S. B. Raredon, J. Dengelegi, K.-Y. Kim, P. Sun, M. Zhong, S. Lee, P. Patra, F. Hyder, L. E. Niklason, S.-H. Lee, Y.-S. Yoon, I.-H. Park, Engineering of human brain organoids with a functional vascular-like system. *Nat. Methods* **16**, 1169–1175 (2019).

10. I. Chiaradia, M. A. Lancaster, Brain organoids for the study of human neurobiology at the interface of in vitro and in vivo. *Nat. Neurosci.* **23**, 1496–1508 (2020).

11. W. A. Anderson, A. Bosak, H. T. Hogberg, T. Hartung, M. J. Moore, Advances in 3D neuronal microphysiological systems: Towards a functional nervous system on a chip. *In Vitro Cell. Dev. Biol. Anim.* **57**, 191–206 (2021).

12. C. A. Thomas Jr, P. A. Springer, G. E. Loeb, Y. Berwald-Netter, L. M. Okun, A miniature microelectrode array to monitor the bioelectric activity of cultured cells. *Exp. Cell Res.* **74**, 61–66 (1972).

13. J. Pine, Recording action potentials from cultured neurons with extracellular microcircuit electrodes. *J. Neurosci. Methods* **2**, 19–31 (1980).

14. M. E. Spira, A. Hai, Multi-electrode array technologies for neuroscience and cardiology. *Nat. Nanotechnol.* **8**, 83–94 (2013).

15. M. Durens, J. Nestor, M. Williams, K. Herold, R. F. Niescier, J. W. Lunden, A. W. Phillips, Y.-C. Lin, D. M. Dykxhoorn, M. W. Nestor, High-throughput screening of human induced pluripotent stem cell-derived brain organoids. *J. Neurosci. Methods* **335**, 108627 (2020).

16. A. Hai, A. Dormann, J. Shappir, S. Yitzchaik, C. Bartic, G. Borghs, J. P. M. Langedijk, M. E. Spira, Spine-shaped gold protrusions improve the adherence and electrical coupling of neurons with the surface of micro-electronic devices. *J. R. Soc. Interface* **6**, 1153–1165 (2009).

17. J. T. Robinson, M. Jorgolli, A. K. Shalek, M.-H. Yoon, R. S. Gertner, H. Park, Vertical nanowire electrode arrays as a scalable platform for intracellular interfacing to neuronal circuits. *Nat. Nanotechnol.* **7**, 180–184 (2012).

18. J. Abbott, T. Ye, L. Qin, M. Jorgolli, R. S. Gertner, D. Ham, H. Park, CMOS nanoelectrode array for all-electrical intracellular electrophysiological imaging. *Nat. Nanotechnol.* **12**, 460–466 (2017).

19. Y. Liu, A. F. McGuire, H.-Y. Lou, T. L. Li, J. B.-H. Tok, B. Cui, Z. Bao, Soft conductive micropillar electrode arrays for biologically relevant electrophysiological recording. *Proc. Natl. Acad. Sci. U.S.A.* **115**, 11718–11723 (2018).

20. J. Cools, Q. Jin, E. Yoon, D. Alba Burbano, Z. Luo, D. Cuypers, G. Callewaert, D. Braeken, D. H. Gracias, A micropatterned multielectrode shell for 3D spatiotemporal recording from live cells. *Adv. Sci.* **5**, 1700731 (2018).

21. A. Kalmykov, C. Huang, J. Bliley, D. Shiwarski, J. Tashman, A. Abdullah, S. K. Rastogi, S. Shukla, E. Mataev, A. W. Feinberg, K. J. Hsia, T. Cohen-Karni, Organ-on-e-chip: Three-dimensional self-rolled biosensor array for electrical interrogations of human electrogenic spheroids. *Sci. Adv.* **5**, eaax0729 (2019).

22. D. A. Soscia, D. Lam, A. C. Tooker, H. A. Enright, M. Triplett, P. Karande, S. K. G. Peters, A. P. Sales, E. K. Wheeler, N. O. Fischer, A flexible 3-dimensional microelectrode array for in vitro brain models. *Lab Chip* **20**, 901–911 (2020).

23. A. Kalmykov, J. W. Reddy, E. Bedoyan, Y. Wang, R. Garg, S. K. Rastogi, D. Cohen-Karni, M. Chamanzar, T. Cohen-Karni, Bioelectrical interfaces with cortical spheroids in three-dimensions. *J. Neural Eng.* **18**, 055005 (2021).

24. Y. Park, C. K. Franz, H. Ryu, H. Luan, K. Y. Cotton, J. U. Kim, T. S. Chung, S. Zhao, A. Vazquez-Guardado, D. S. Yang, K. Li, R. Avila, J. K. Phillips, M. J. Quezada, H. Jang, S. S. Kwak, S. M. Won, K. Kwon, H. Jeong, A. J. Bandodkar, M. Han, H. Zhao, G. R. Osher, H. Wang, K. Lee, Y. Zhang, Y. Huang, J. D. Finan, J. A. Rogers, Three-dimensional, multifunctional neural interfaces for cortical spheroids and engineered assembloids. *Sci. Adv.* **7**, eabf9153 (2021).

25. Y. Park, T. S. Chung, J. A. Rogers, Three dimensional bioelectronic interfaces to small-scale biological systems. *Curr. Opin. Biotechnol.* **72**, 1–7 (2021).

26. P. Le Floch, Q. Li, Z. Lin, S. Zhao, R. Liu, K. Tasnim, H. Jiang, J. Liu, Stretchable mesh nanoelectronics for 3D single-cell chronic electrophysiology from developing brain organoids. *Adv. Mater.* **34**, e2106829 (2022).

27. Q. Huang, T. Deng, W. Xu, C. K. Yoon, Z. Qin, Y. Lin, T. Li, Y. Yang, M. Shen, S. M. Thon, J. B. Khurgin, D. H. Gracias, Solvent responsive self-folding of 3D photosensitive graphene architectures. *Adv. Intel. Sys.* 2000195 (2020).

28. C.-T. Lin, L.-W. Ko, M.-H. Chang, J.-R. Duann, J.-Y. Chen, T.-P. Su, T.-P. Jung, Review of wireless and wearable electroencephalogram systems and brain-computer interfaces—A mini-review. *Gerontology* **56**, 112–119 (2010).

29. K. V. Nemani, K. L. Moodie, J. B. Brennick, A. Su, B. Gimi, In vitro and in vivo evaluation of SU8 biocompatibility. *Mater. Sci. Eng. C* **33**, 4453–4459 (2013).

30. A.-N. Cho, Y. Jin, Y. An, J. Kim, Y. S. Choi, J. S. Lee, J. Kim, W.-Y. Choi, D.-J. Koo, W. Yu, G.-E. Chang, D.-Y. Kim, S.-H. Jo, J. Kim, S.-Y. Kim, Y.-G. Kim, J. Y. Kim, N. Choi, E. Cheong, Y.-J. Kim, H. S. Je, H.-C. Kang, S.-W. Cho, Microfluidic device with brain extracellular matrix promotes structural and functional maturation of human brain organoids. *Nat. Commun.* **12**, 4730 (2021).

31. J. P. Neto, P. Baião, G. Lopes, J. Frazão, J. Nogueira, E. Fortunato, P. Barquinha, A. R. Kampff, Does Impedance matter when recording spikes with polytrodes? *Front. Neurosci.* **12**, 715 (2018).

32. M. Chesnut, T. Hartung, H. Hogberg, D. Pamies, Human oligodendrocytes and myelin in vitro to evaluate developmental neurotoxicity. *Int. J. Mol. Sci.* **22**, 7929 (2021).

33. M. Chesnut, H. Paschoud, C. Repond, L. Smirnova, T. Hartung, M. G. Zurich, H. T. Hogberg, D. Pamies, Human ipsc-derived model to study myelin disruption. *Int. J. Mol. Sci.* **22**, 9473 (2021).

34. H. Renner, M. Grabos, K. J. Becker, T. E. Kagermeier, J. Wu, M. Otto, S. Peischard, D. Zeuschner, Y. TsyTsyura, P. Disse, J. Klingauf, S. A. Leidel, G. Seebohm, H. R. Schöler, J. M. Bruder, A fully

automated high-throughput workflow for 3D-based chemical screening in human midbrain organoids. *eLife* **9**, e52904 (2020).

35. H. G. Rey, C. Pedreira, R. Quian Quiroga, Past, present and future of spike sorting techniques. *Brain Res. Bull.* **119**, 106–117 (2015).

36. M. S. Lewicki, A review of methods for spike sorting: The detection and classification of neural action potentials. *Network* **9**, R53–R78 (1998).

37. Y. Han, H. Zhu, Y. Zhao, Y. Lang, H. Sun, J. Han, L. Wang, C. Wang, J. Zhou, The effect of acute glutamate treatment on the functional connectivity and network topology of cortical cultures. *Med. Eng. Phys.* **71**, 91–97 (2019).

38. A. I. McLeod, Kendall rank correlation and Mann-Kendall trend test. *R Package Kendall* (2005; https://cran.r-project.org/web/packages/Kendall/index.html).

39. S. P. Lacour, G. Courtine, J. Guck, Materials and technologies for soft implantable neuroprostheses. *Nat. Rev. Mater.* **1**, 16063 (2016)

40. O. Erol, A. Pantula, W. Liu, D. H. Gracias, Transformer hydrogels: A review. *Adv. Mater. Technol.* **4**, 1900043 (2019).

41. N. Brandenberg, S. Hoehnel, F. Kuttler, K. Homicsko, C. Ceroni, T. Ringel, N. Gjorevski, G. Schwank, G. Coukos, G. Turcatti, M. P. Lutolf, High-throughput automated organoid culture via stem-cell aggregation in microcavity arrays. *Nat. Biomed. Eng.* **4**, 863–874 (2020).

42. M. Antonijevic, M. Zivkovic, S. Arsic, A. Jevremovic, Using AI-based classification techniques to process EEG data collected during the visual short-term memory assessment. *J. Sensors.* **2020**, 1–12 (2020).

43. M. Golmohammadi, A. H. H. N. Torbati, S. L. de Diego, I. Obeid, J. Picone, Automatic analysis of EEGs using big data and hybrid deep learning architectures. *Front. Hum. Neurosci.* **13**, 76 (2019).

44. S. Modafferi, X. Zhong, A. Kleensang, Y. Murata, F. Fagiani, D. Pamies, H. T. Hogberg, V. Calabrese, H. Lachman, T. Hartung, L. Smirnova, Gene-environment interactions in developmental neurotoxicity: A case study of synergy between chlorpyrifos and CHD8 knockout in human BrainSpheres. *Environ. Health Perspect.* **129**, 077001 (2021).

45. K. Chen, Y. Jiang, Z. Wu, N. Zheng, H. Wang, H. Hong, HTsort: Enabling fast and accurate spike sorting on multi-electrode arrays. *Front. Comput. Neurosci.* **15**, 657151 (2021).

46. C. Rossant, S. N. Kadir, D. F. M. Goodman, J. Schulman, M. L. D. Hunter, A. B. Saleem, A. Grosmark, M. Belluscio, G. H. Denfield, A. S. Ecker, A. S. Tolias, S. Solomon, G. Buzsaki, M. Carandini, K. D. Harris, Spike sorting for large, dense electrode arrays. *Nat. Neurosci.* **19**, 634–641 (2016).

47. S. Yang, J. Qu, Computing thermomechanical properties of crosslinked epoxy by molecular dynamic simulations. *Polymer* **53**, 4806–4817 (2012).

48. A. Bandyopadhyay, P. K. Valavala, T. C. Clancy, K. E. Wise, G. M. Odegard, Molecular modeling of crosslinked epoxy polymers: The effect of crosslink density on thermomechanical properties. *Polymer* **52**, 2445–2452 (2011).

49. N. R. Langley, K. E. Polmanteer, Relation of elastic modulus to crosslink and entanglement concentrations in rubber networks. *J. Polym. Sci. Polym. Phys. Ed.* **12**, 1023–1034 (1974).
